# Supplementary material for: Aluminous phyllosilicates promote exceptional nanoscale preservation of biogeochemical heterogeneities in Archaean siliciclastic microbial mats
Source: Nat Commun. 2025 Mar 19;16:2726. doi: 10.1038/s41467-025-57727-4 (PMC11923192; doi:10.1038/s41467-025-57727-4)
Supplement: Supplementary file 1 — Supplementary Information [file 41467_2025_57727_MOESM1_ESM.pdf]

Supplementary Data for "Aluminous phyllosilicates promote exceptional nanoscale preservation of biogeochemical heterogeneities in Archaean siliciclastic microbial mats"

Keyron Hickman-Lewis[1,2]*, Javier Cuadros[3], Keewook Yi[4], Tae Eun Hong[5], Mirang Byeon[5], Jae Hyuck Jang[6], Min-Yeong Choi[6,7], YoonKyung Seo[6,8], Jens Najorka[3], Wren Montgomery[3], Krzysztof Matlak[9], Barbara Wolanin[9], Caroline L. Smith[3,10], Barbara Cavalazzi[11,12]

[1] School of Natural Sciences, Birkbeck, University of London, Malet Street, Bloomsbury, London, WC1E 7HX, United Kingdom
[2] Department of Earth Science and Engineering, Imperial College London, London, SW7 2BX, United Kingdom
[3] Natural History Museum, Cromwell Road, London, SW7 5BD, United Kingdom
[4] Korea Basic Science Institute, 162, Yeongudanji-ro, Ochang-eup, Cheongwon-gu, Cheongju-si, Chungcheongbuk-do, 28119, Republic of Korea
[5] Korea Basic Science Institute, 60, Gwahaksandan 1-ro, Gangseo-gu, Busan, Republic of Korea
[6] Korea Basic Science Institute, 169-148, Gwahak-ro, Yuseong-gu, Daejeon, Republic of Korea
[7] Sungkyunkwan University, 2066, Seobu-ro, Jangan-gu, Suwon, Republic of Korea
[8] National NanoFab Center, 291 Daehak-ro, Yuseong-gu, Daejeon, 34141, Republic of Korea
[9] SOLARIS, National Synchrotron Radiation Centre, Jagiellonian University, Czerwone Maki 98, 30-392, Kraków, Poland
[10] School of Geographical and Earth Sciences, University of Glasgow, Glasgow G12 8QQ, United Kingdom
[11] Dipartimento di Scienze Biologiche, Geologiche e Ambientali, Università di Bologna, via Zamboni 67, I-40126 Bologna, Italy
[12] Department of Geology, University of Johannesburg, PO Box 524, Auckland Park 2006, Johannesburg, South Africa

*correspondence: k.hickman-lewis@bbk.ac.uk

Geological setting

The samples studied were collected by the Archaean Biosphere Drilling Project (ABDP). Our samples come from ABDP core #5, through the Mosquito Creek Formation (MCF) in Mosquito Creek Basin (Pilbara craton), collected at 21°41′53.7″S, 120°37′14.5″E in the Eastern Creek area northeast of the town of Nullagine (Bagas et al., 2008). The age of the horizons cored is constrained to between 2971 ± 15 Ma and 2905 ± 9 Ma (Thorpe et al., 1992; Bagas et al., 2008). Optical petrography shown herein indicates that the sandstones studied are organic-poor relative to the shales studied by Koehler et al. (2019); for this reason, we avoided whole-rock approaches and conducted an *in situ* multi-technique analytical strategy to decode the biogeochemistry of microbial laminations. For full details of the stratigraphy and palaeoenvironmental setting of the MCF, we refer the reader to Hickman (1984), Eriksson et al. (1994), Ohmoto et al. (2006), Bagas et al. (2008) and Nijman et al. (2010).

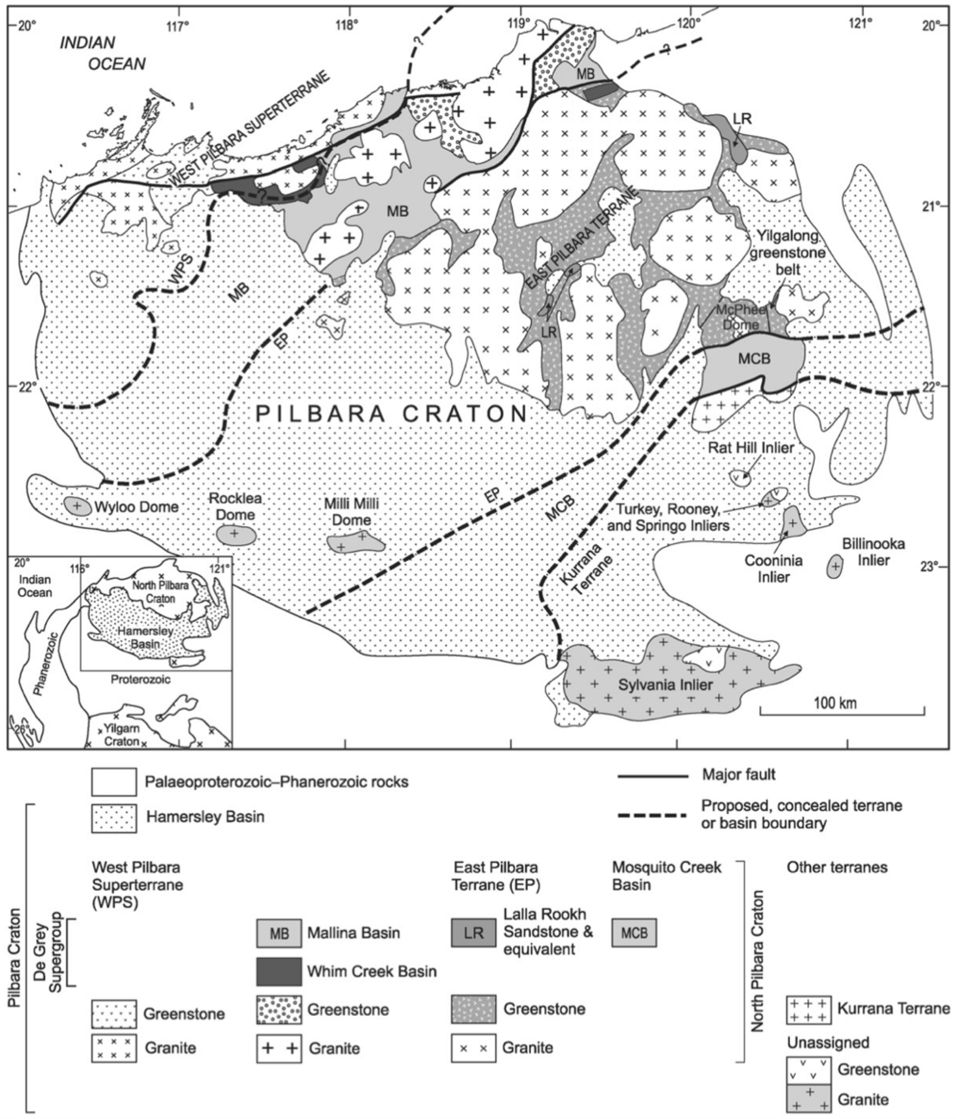

Figure S1. Simplified geological map of the North Pilbara craton. Core ABDP#5 was collected from the Mosquito Creek Basin (MCB). Modified from Bagas et al. (2008).

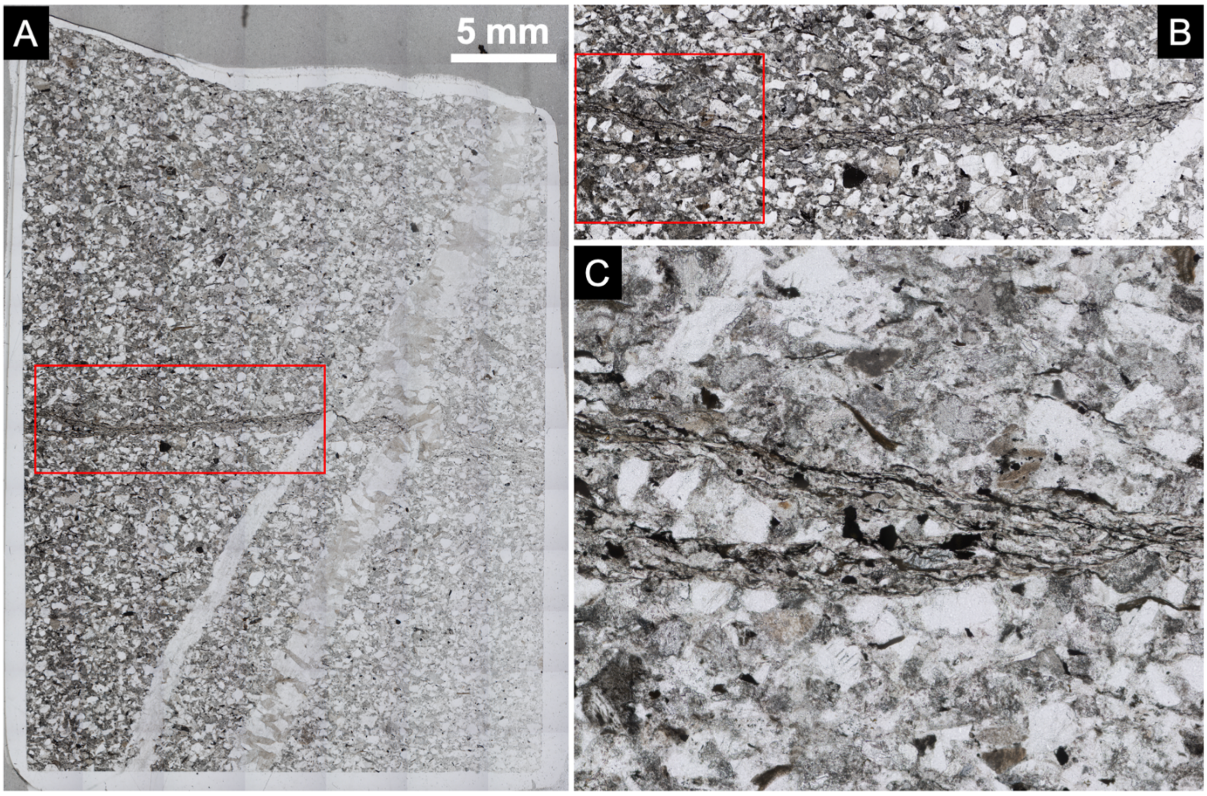

Figure S2. Optical photomicrographs showing thin, organic-poor microbial mat laminations within medium-grade subangular sandstones of the Mosquito Creek Formation. Red box in A shows region imaged in B; red box in B shows region imaged in C. Note bifurcation around zones of sedimentary particles in C. Sample 21a.

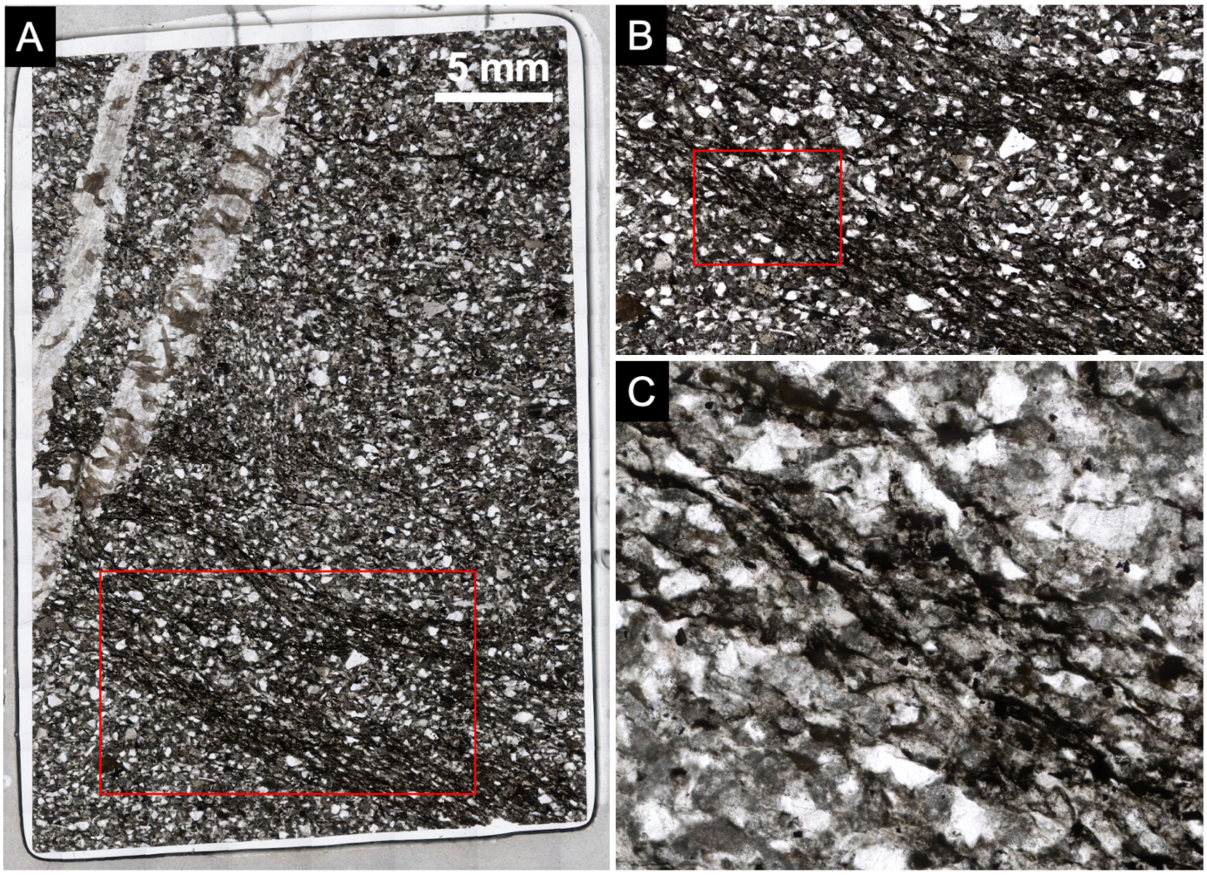

Figure S3. Optical photomicrographs showing undulatory, organic-rich microbial mat laminations within medium-grade subangular sandstones of the Mosquito Creek Formation. Red box in A shows region imaged in B; red box in B shows region imaged in C. Sample 21b.

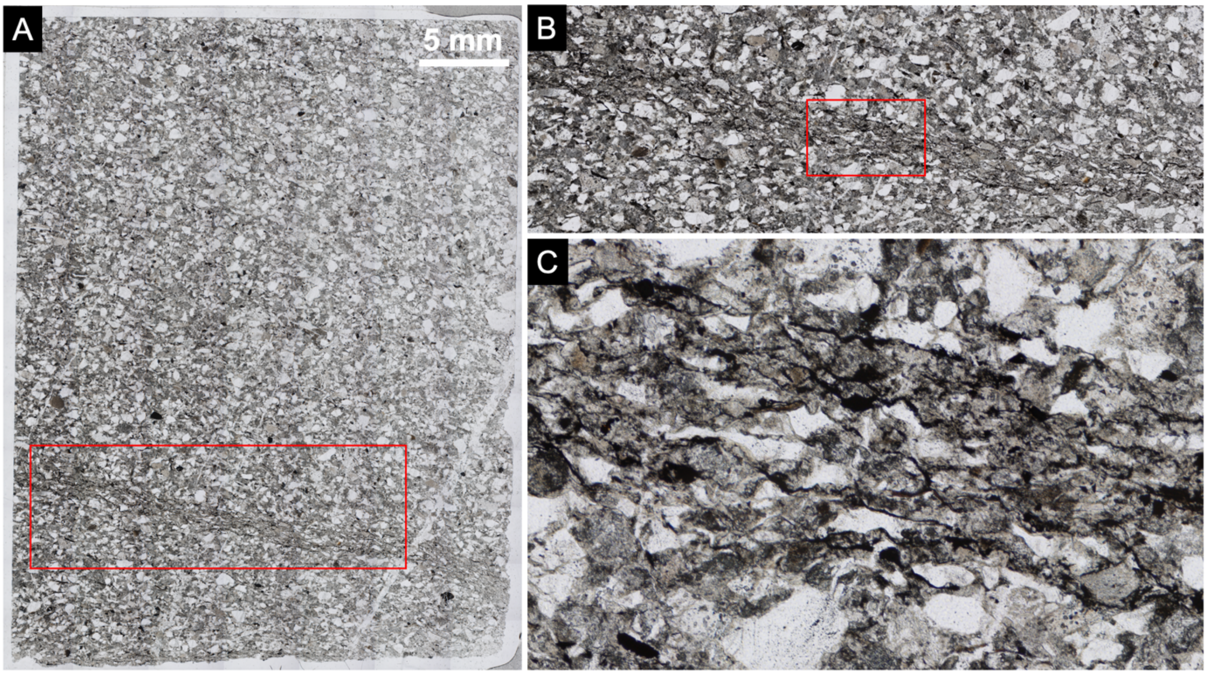

Figure S4. Optical photomicrographs showing thin, organic-poor microbial mat laminations within medium-grade angular sandstones of the Mosquito Creek Formation. Red box in A shows region imaged in B; red box in B shows region imaged in C. Sample 21c.

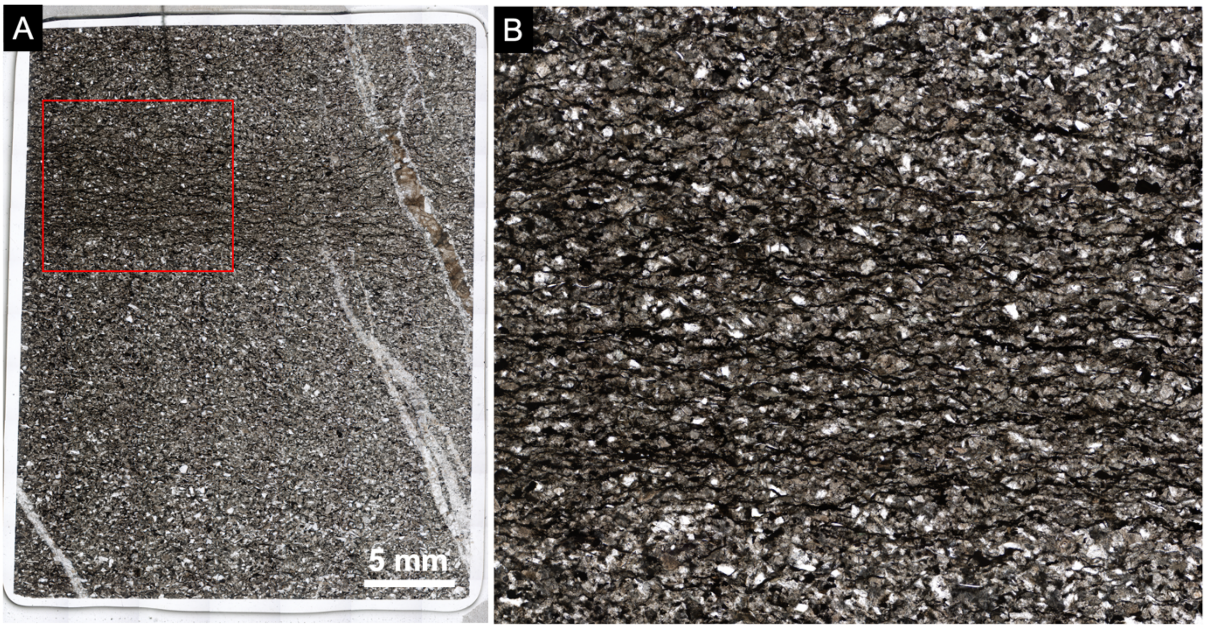

Figure S5. Optical photomicrographs showing thin, flat-laminated microbial mat laminations within fine-grade subangular sandstones of the Mosquito Creek Formation. Red box in A shows region imaged in B. Sample 26d.

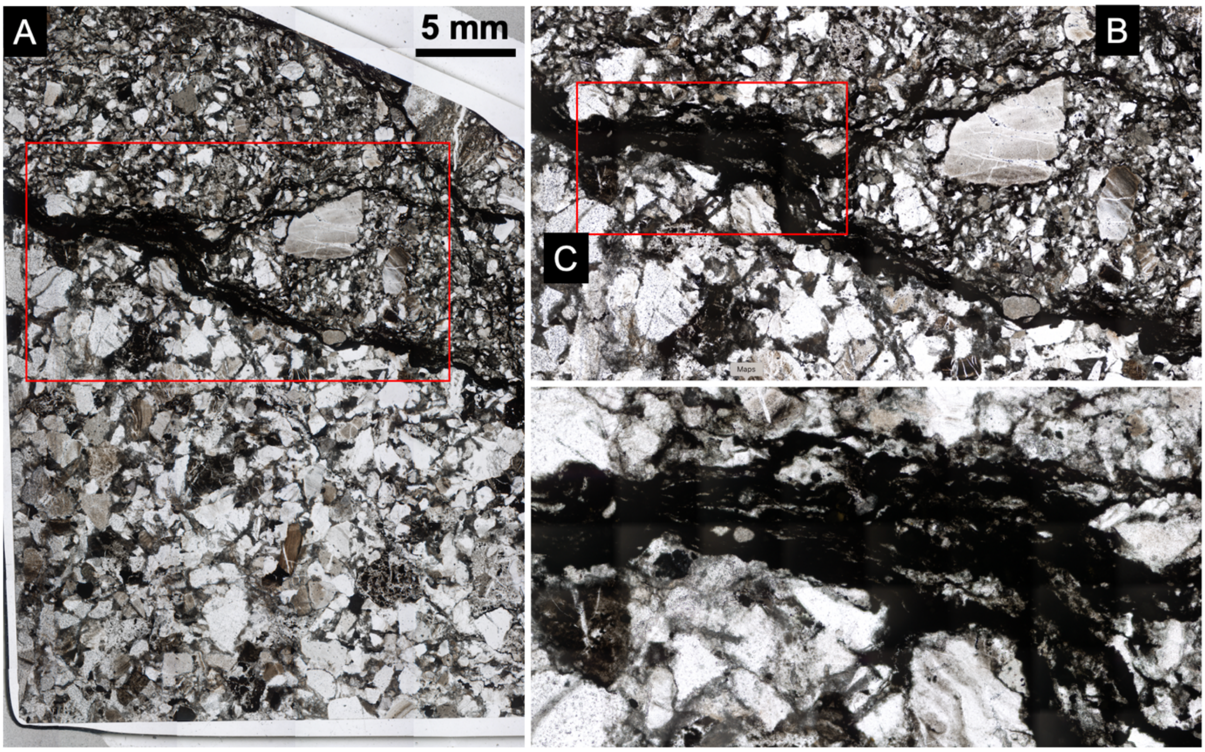

Figure S6. Optical photomicrographs showing exceptionally thick layer of well-laminated microbial mats at the interfacr of very coarse- and coarse-grained angular–subangular sandstones of the Mosquito Creek Formation. Red box in A shows region imaged in B; red box in B shows region imaged in C. Sample 28c.

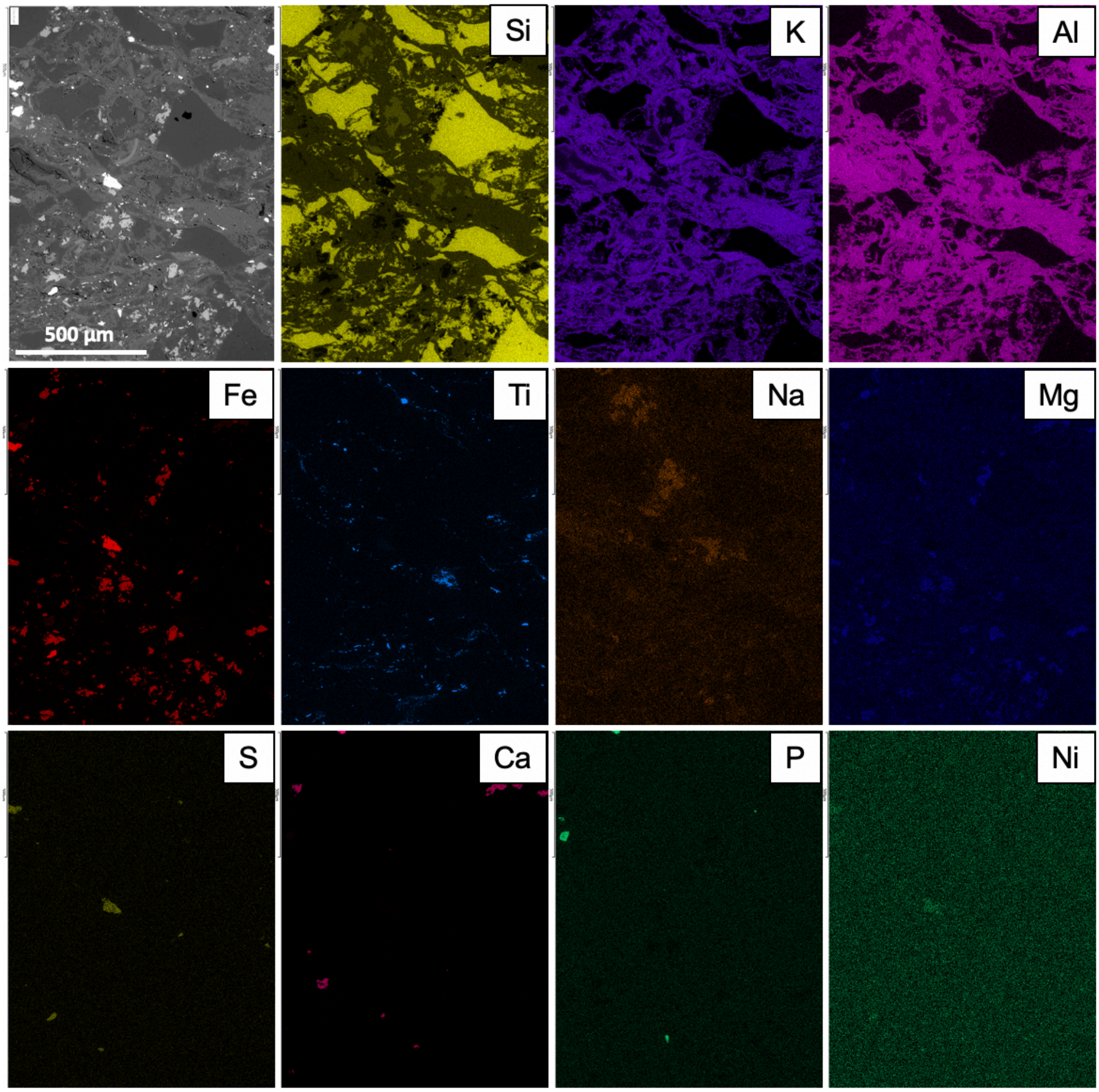

Figure S7. SEM micrograph and EDX element maps obtained within a microbial mat layer. Sample 21b.

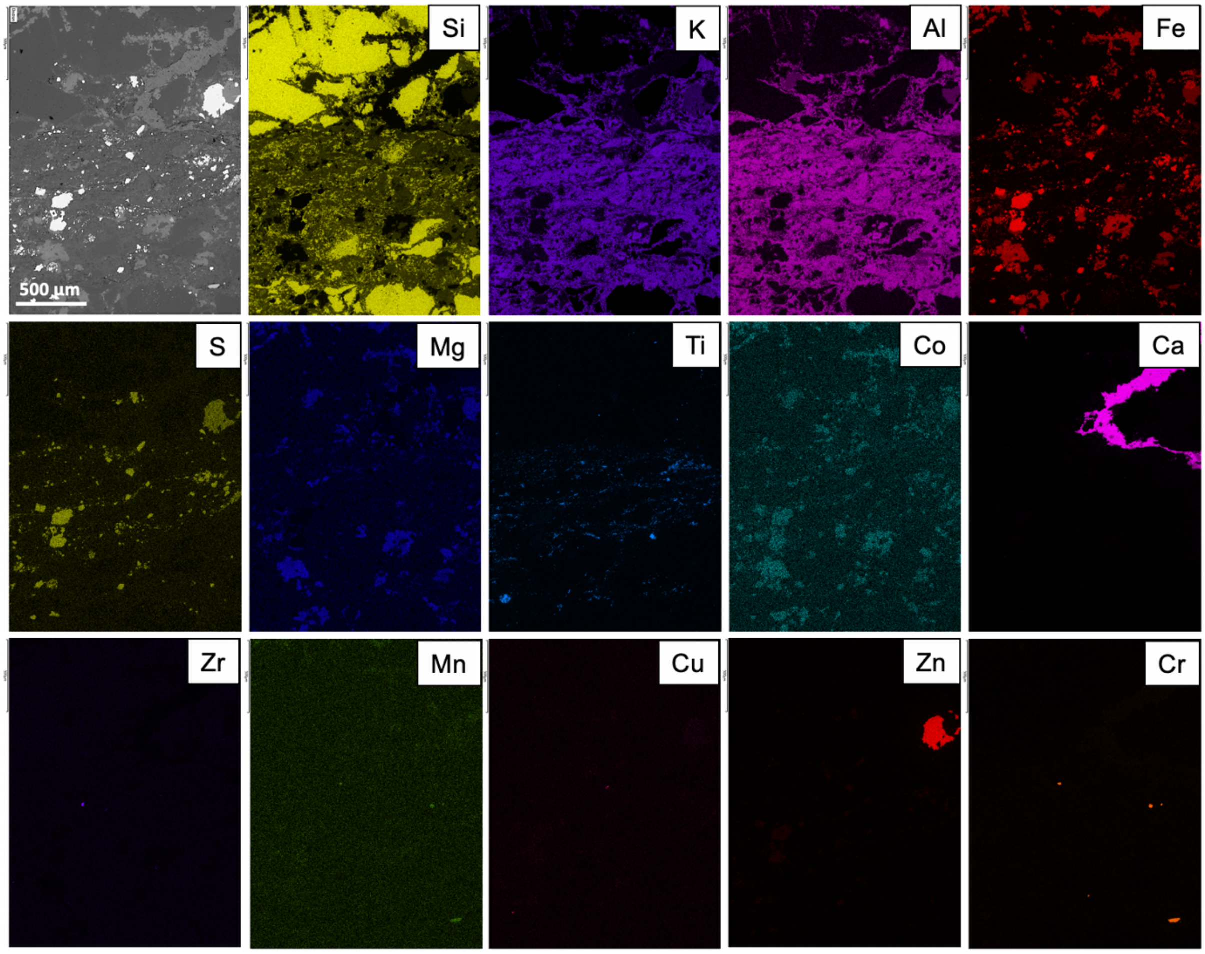

Figure S8. SEM micrograph and EDX element maps showing the transition between a microbial mat layer (lower portion of the region mapped) and the surrounding coarser matrix (upper portion of the region mapped). Sample 28c.

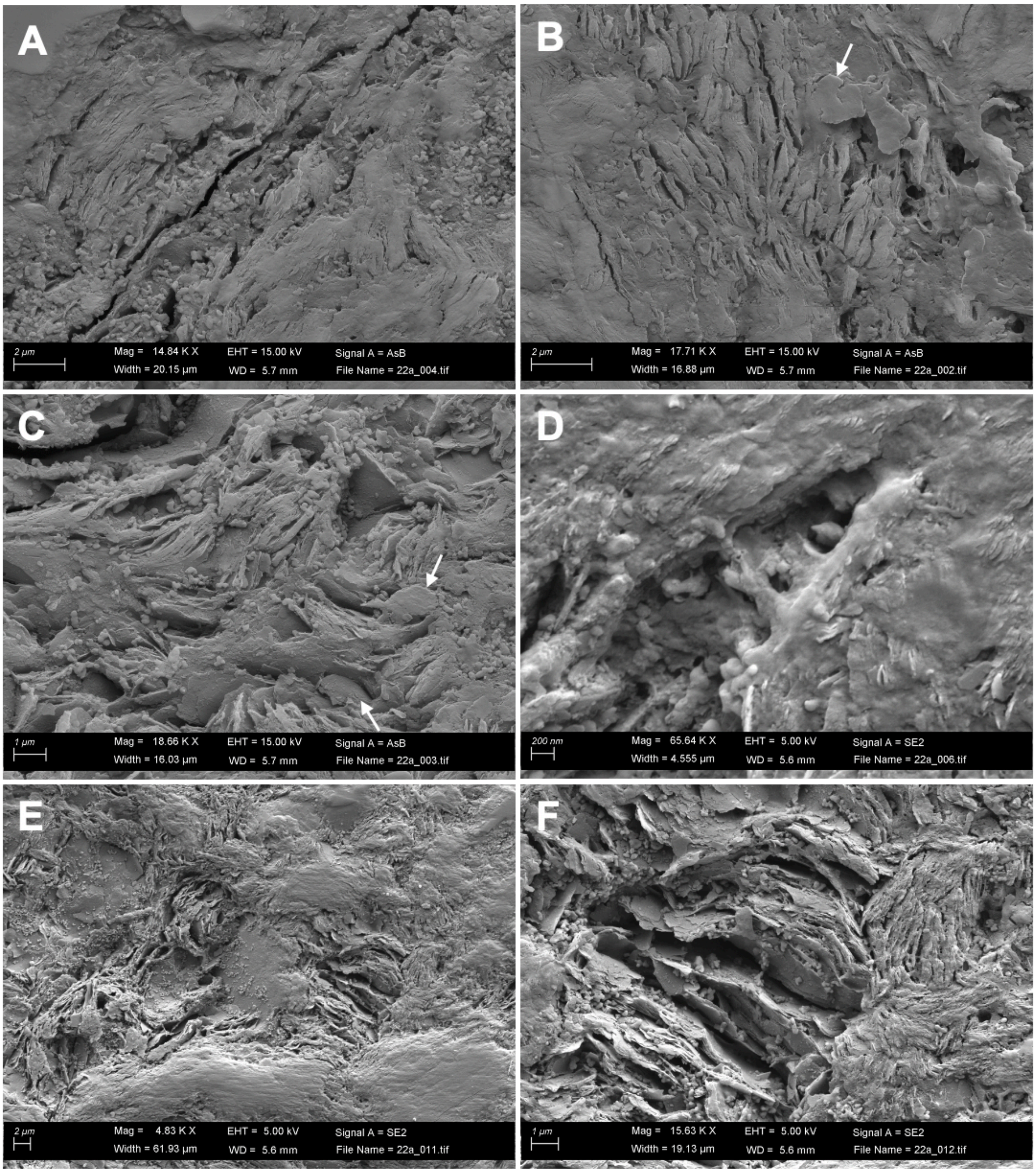

Figure S9. High-magnification SEM micrographs showing phyllosilicate platelets in association with microbial mat-derived organic materials. A) Subaligned platelets approximately parallel to microbial mat layers (NE–SW). B–C) Subaligned platelets showing hexagonal form (examples arrowed). D) Nanometric phyllosilicate platelets embedded within interpreted EPS (amorphous phase). E–F) Oriented domains of illite, interpreted as the product of illitisation of feldspar or other K-bearing phases.

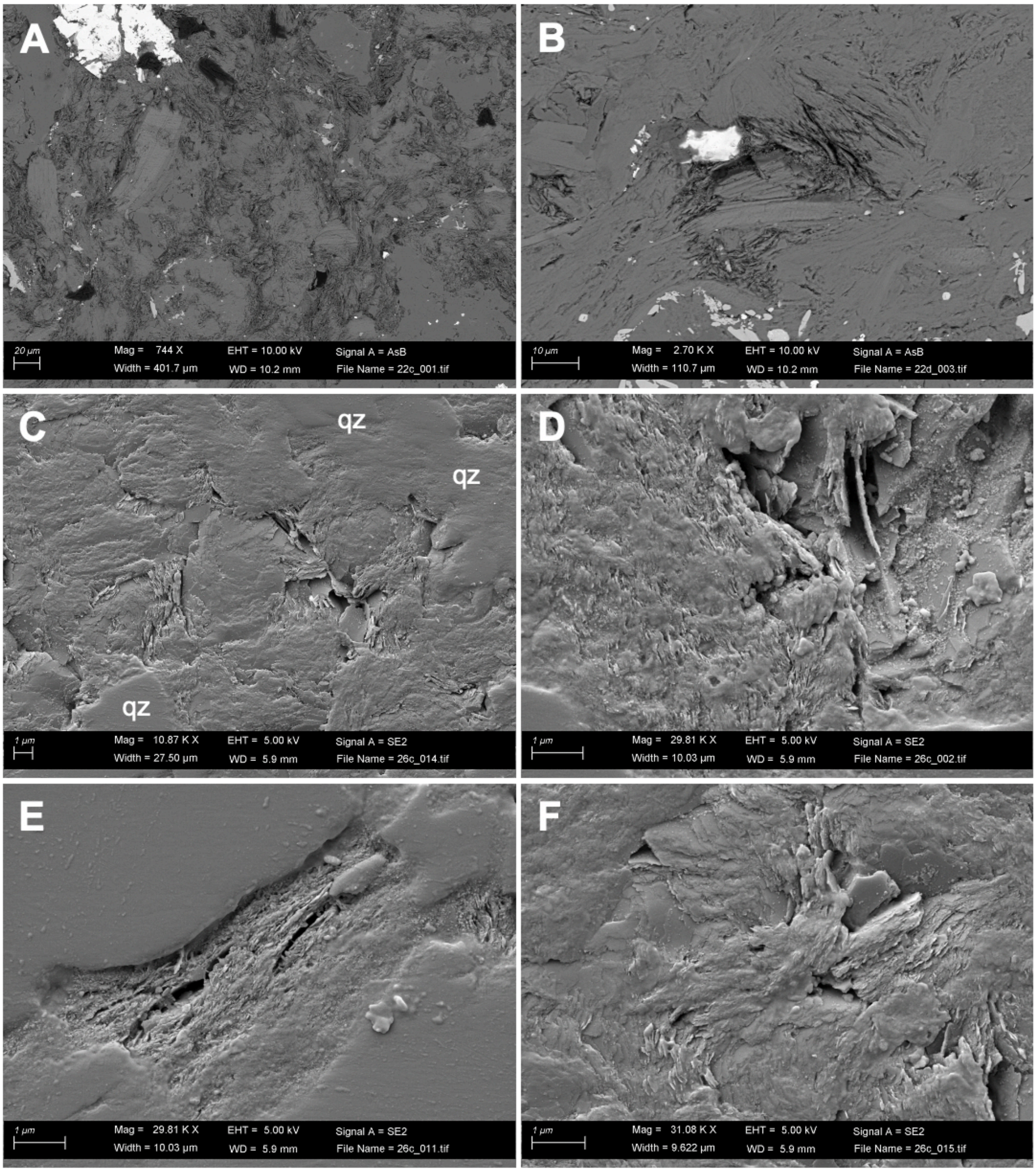

Figure S10. High-magnification SEM micrographs showing phyllosilicate platelets in association with microbial mat-derived organic materials. A) Authigenic and allogenic phyllosilicates associated with microbial mat layers (mat trends broadly vertically). B) Subaligned domains of kaolinite and illite platelets C) Phyllosilicate platelets within the interstices between detrital quartz grains (qz). D–F) Nanometric phyllosilicate platelets associated with microbial mat-derived organic materials.

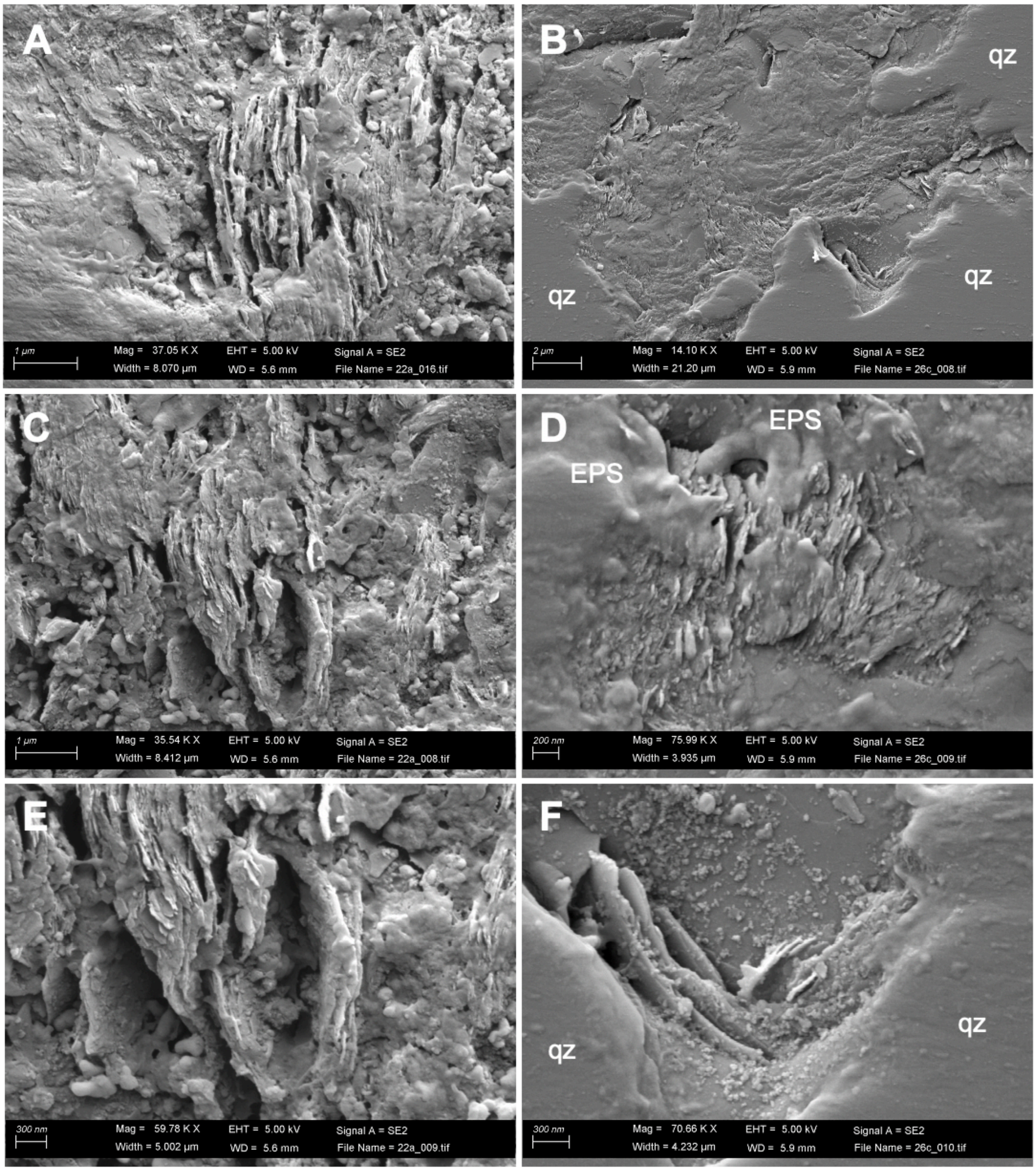

Figure S11. High-magnification SEM micrographs showing phyllosilicate platelets in association with microbial mat-derived organic materials. A,C,E) Authigenic nanometric phyllosilicate platelets associated with microbial mat laminations (mat trends broadly vertically). B) Nanometric phyllosilicate platelets within the interstices between detrital quartz grains (qz). D) Nanometric phyllosilicate platelets associated with interpreted EPS (amorphous organic phase, labelled EPS). F) Phyllosilicate platelets at the edges of quartz (qz) particles, interpreted to show the illitisation of quartz.

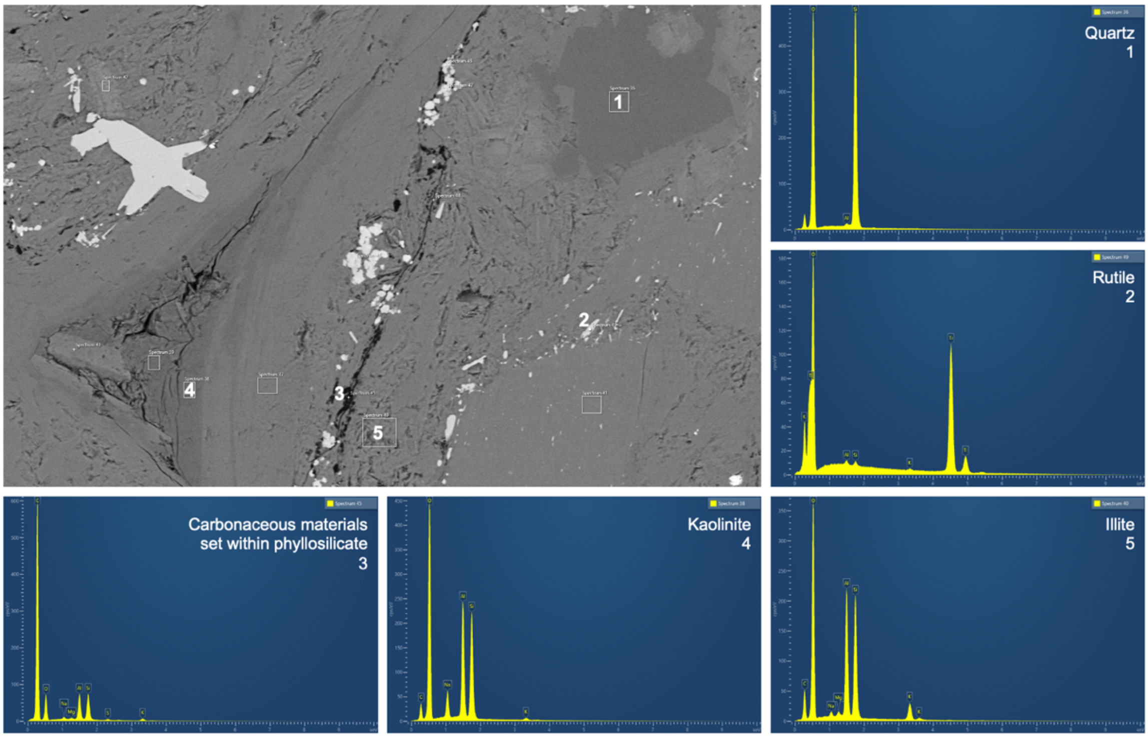

Figure S12. SEM micrograph and EDX spectra. Sample 22d.

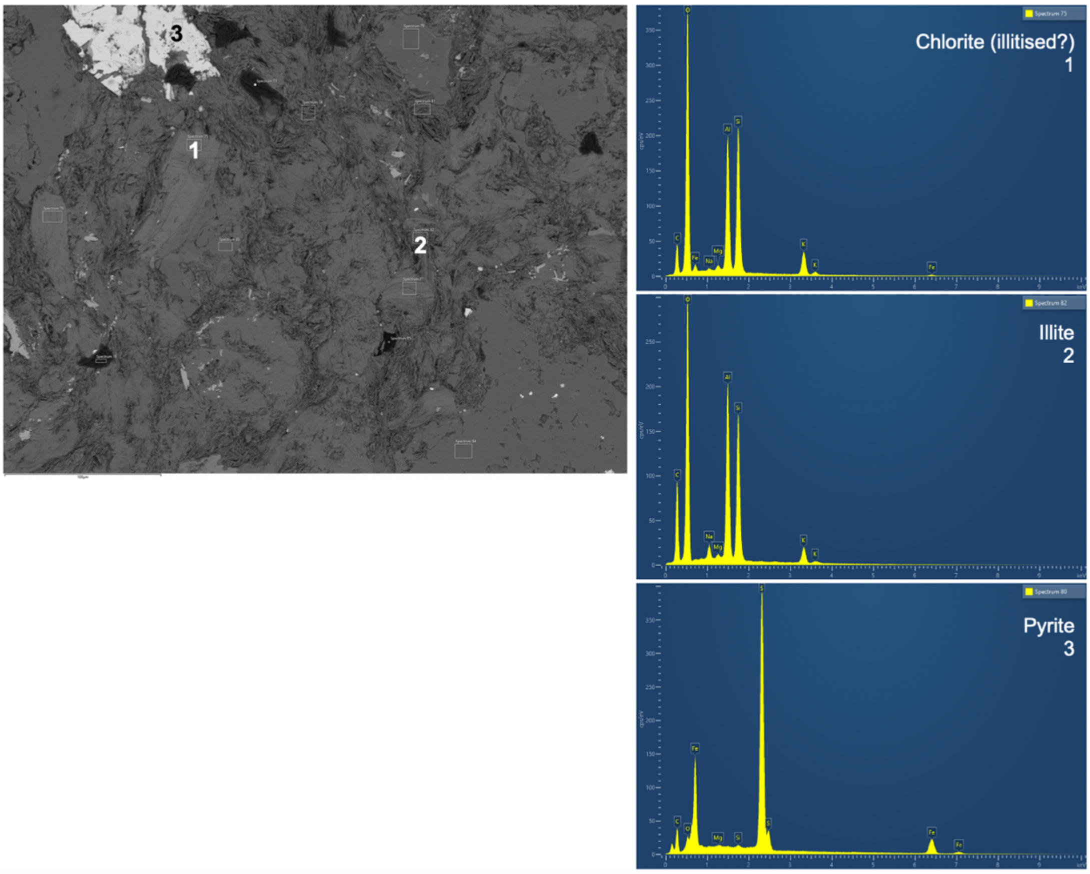

Fig. S13. SEM micrograph and EDX spectra. Sample 22c.

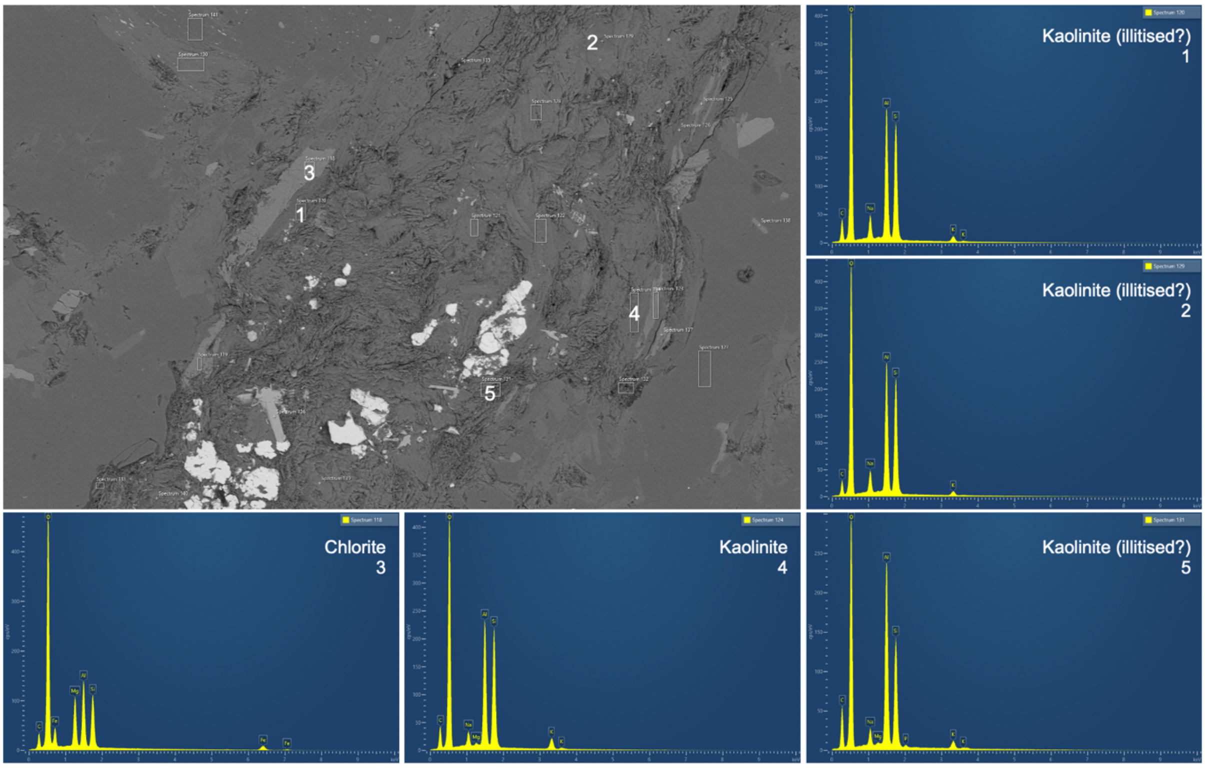

Figure S14. SEM micrograph and EDX spectra. Sample 26b.

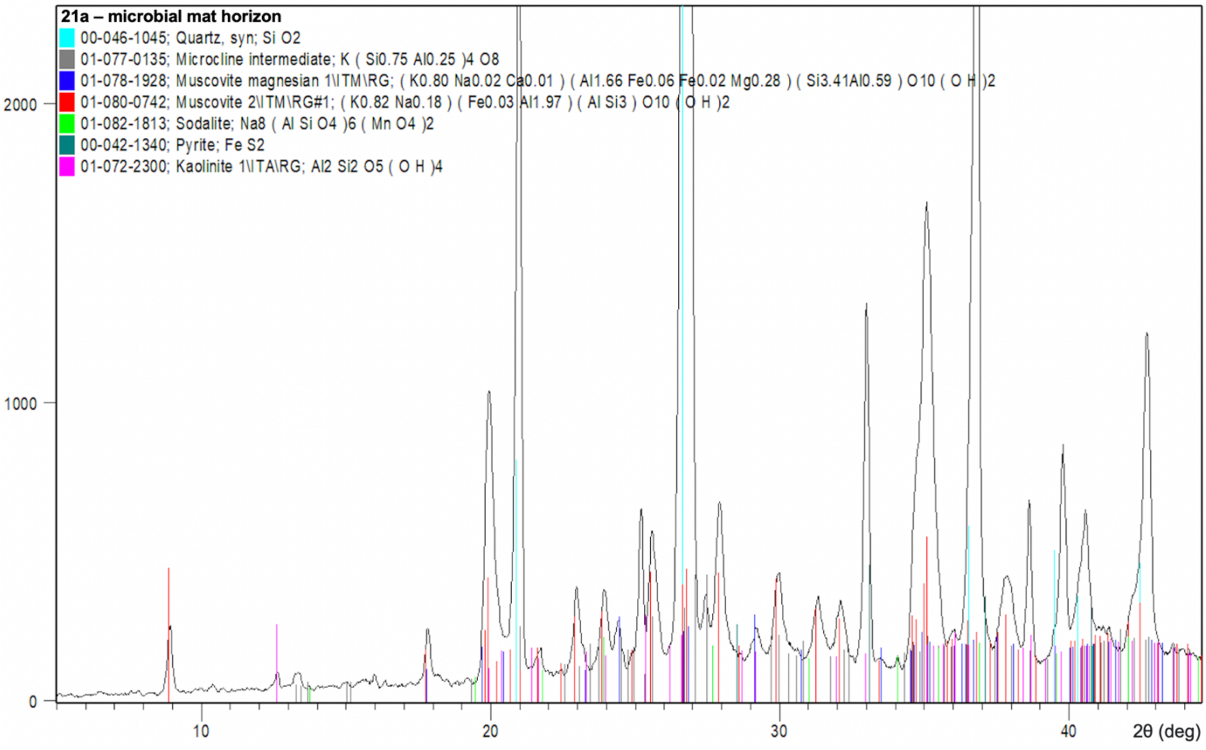

Figure S15. Micro-XRD X-ray diffractogram showing the mineralogical composition of a representative microbial mat horizon. Sample 21a.

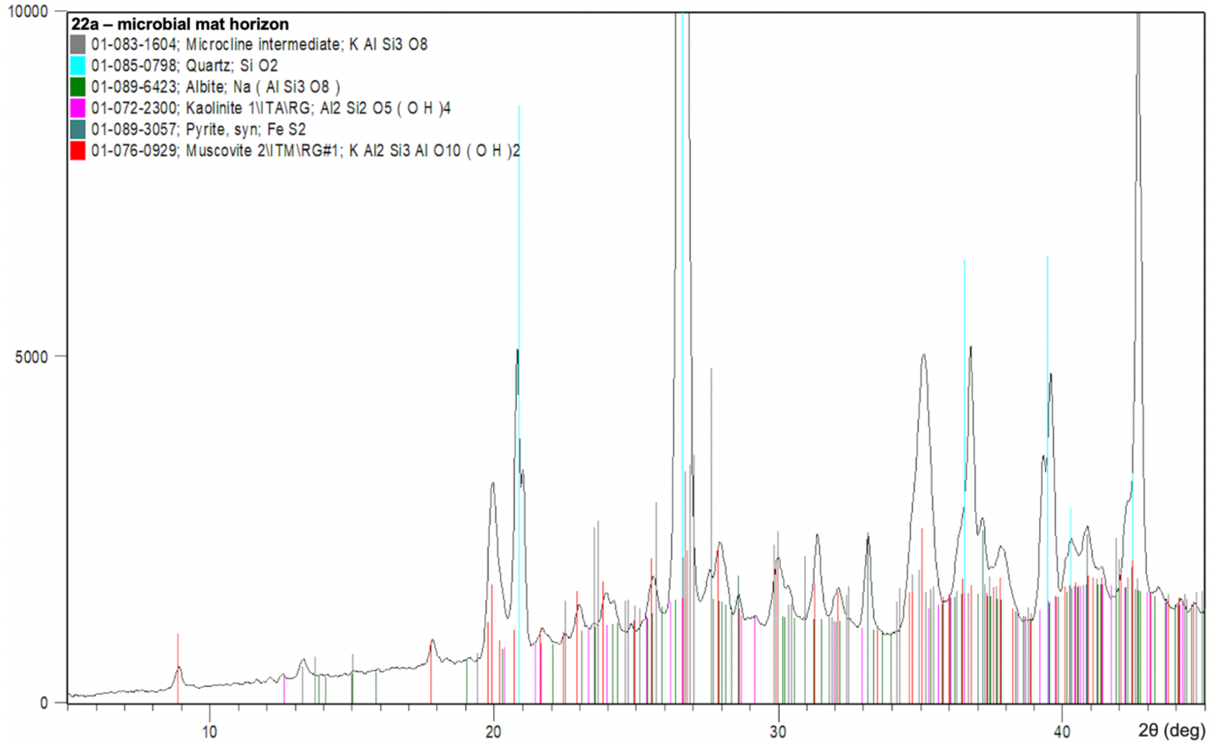

Figure S16. Micro-XRD X-ray diffractogram showing the mineralogical composition of a representative microbial mat horizon. Sample 22a.

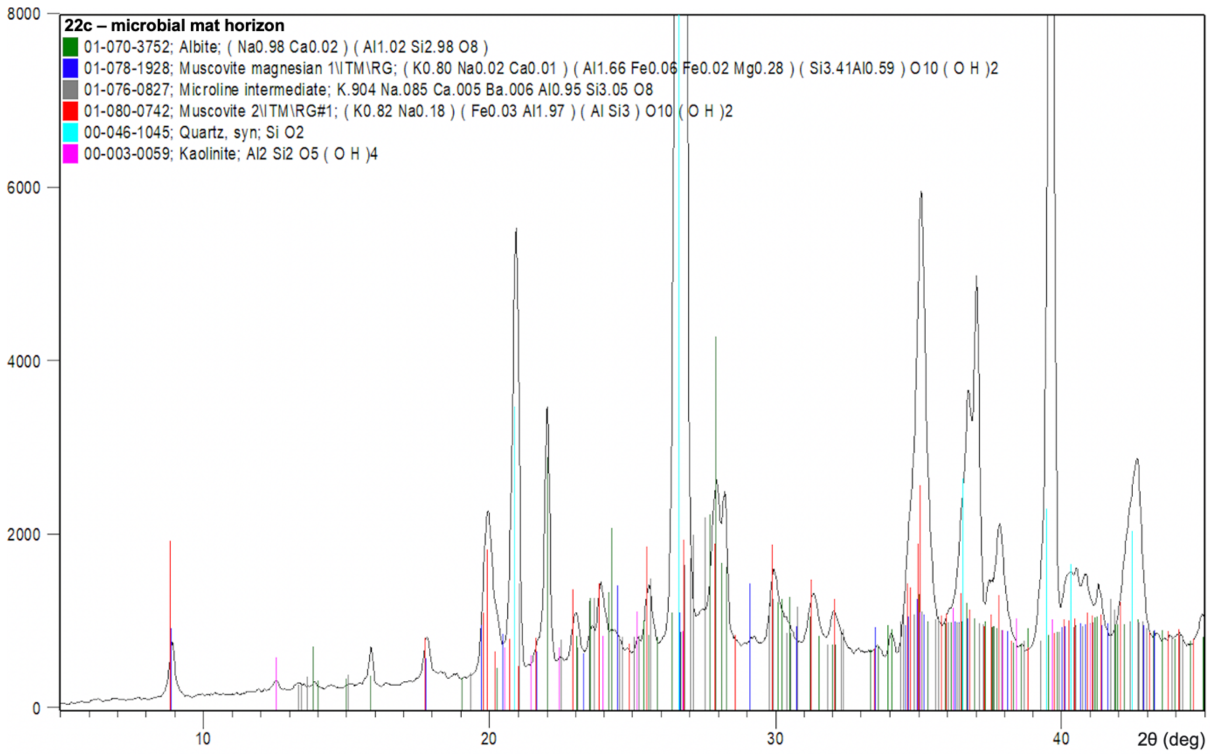

Figure S17. Micro-XRD X-ray diffractogram showing the mineralogical composition of a representative microbial mat horizon. Sample 22c.

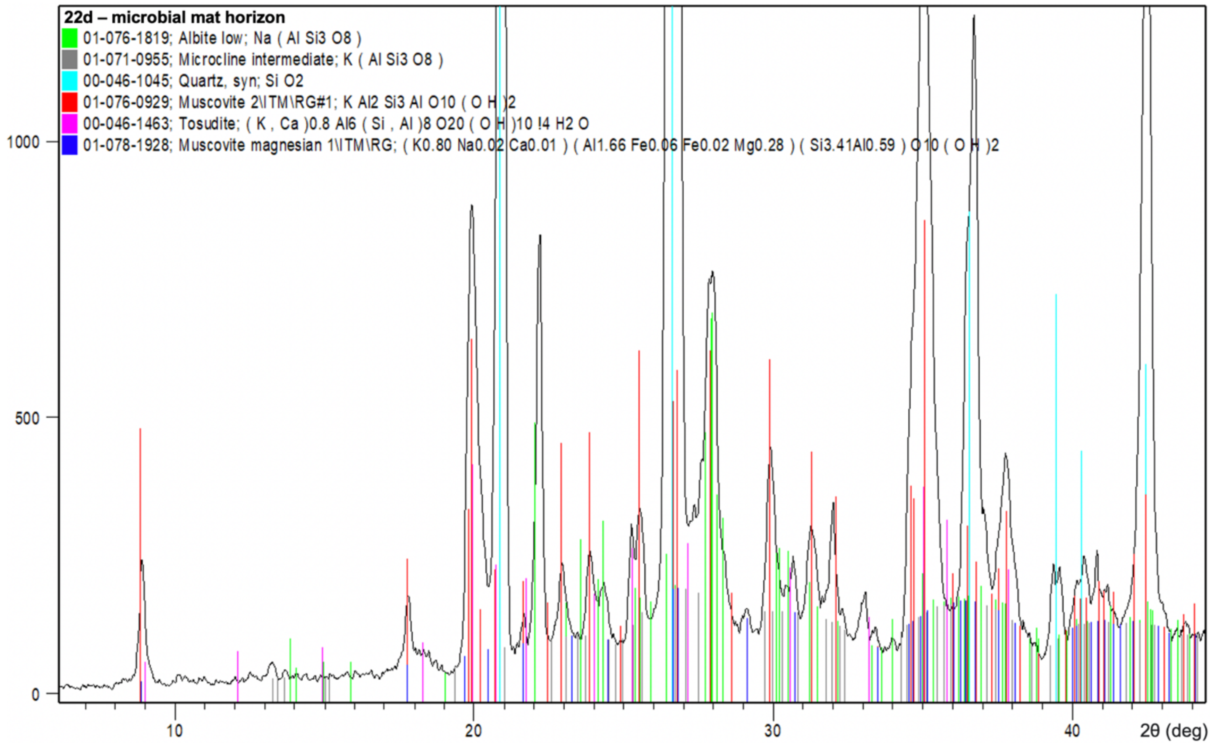

Figure S18. Micro-XRD X-ray diffractogram showing the mineralogical composition of a representative microbial mat horizon. Sample 22d.

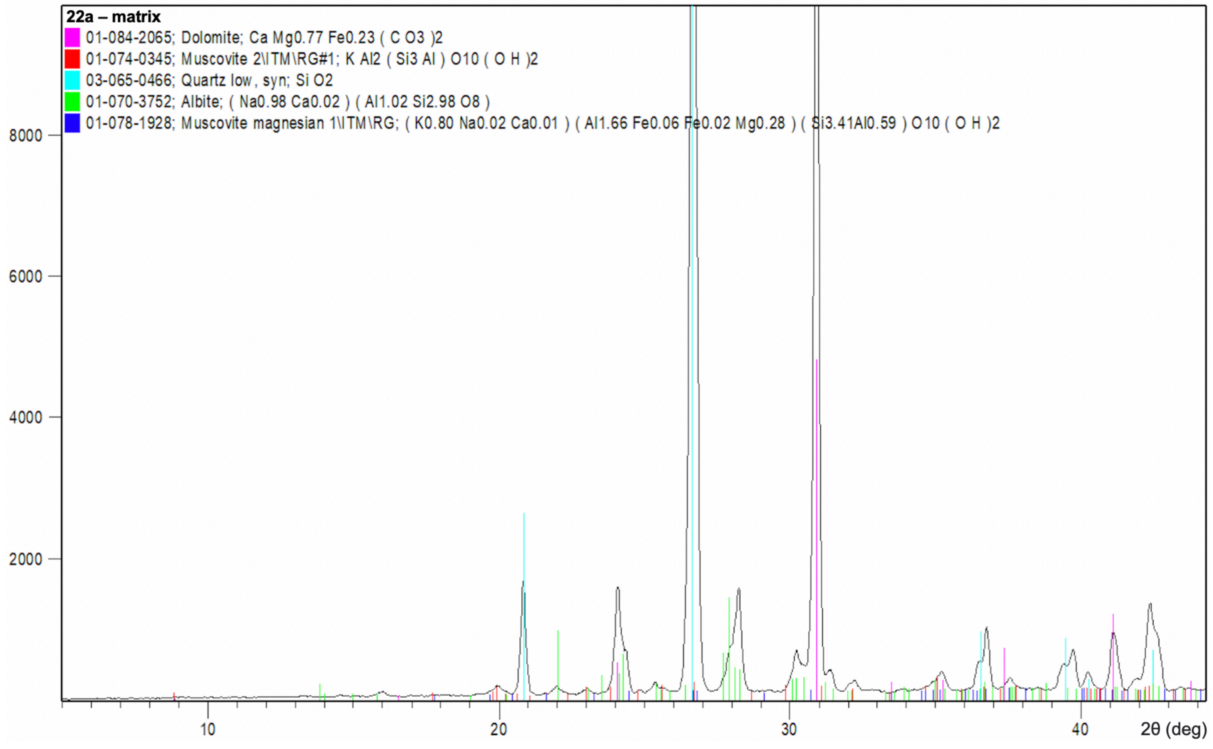

Figure S19. Micro-XRD X-ray diffractogram showing the mineralogical composition of the matrix, i.e. a non-microbial horizon. Sample 22a.

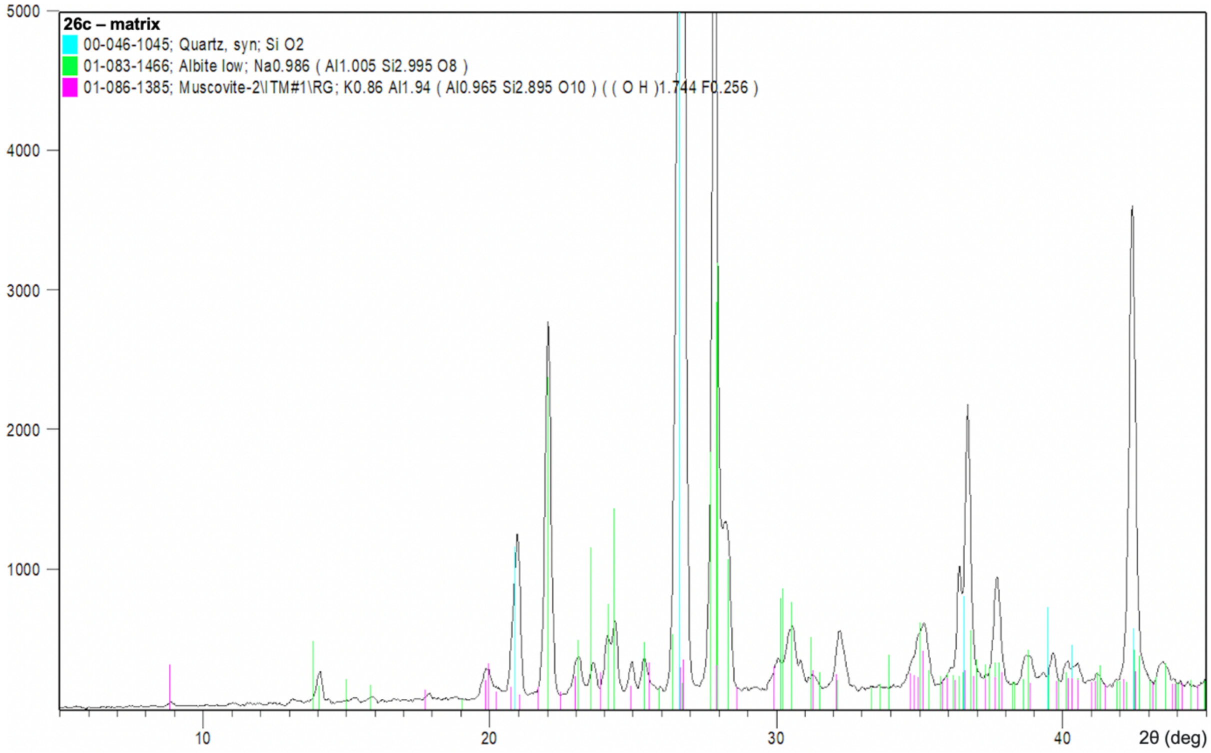

Figure S20. Micro-XRD X-ray diffractogram showing the mineralogical composition of the matrix, i.e. a non-microbial horizon. Sample 26c.

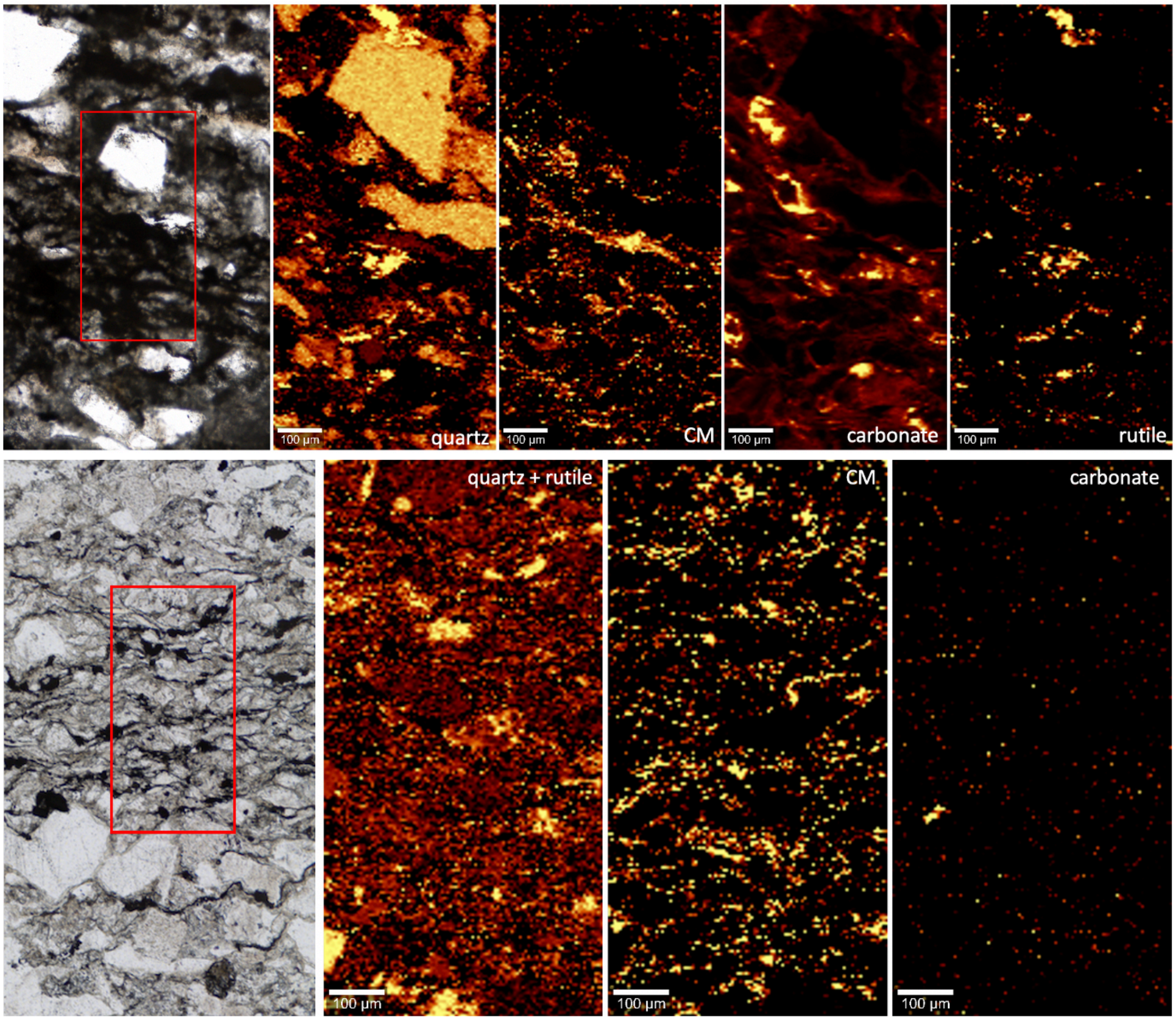

Figure S21. 2D Raman spectral maps showing the distributions of carbonaceous materials and major mineral phases. Row 1 = sample 21b; row 2 = sample 26c.

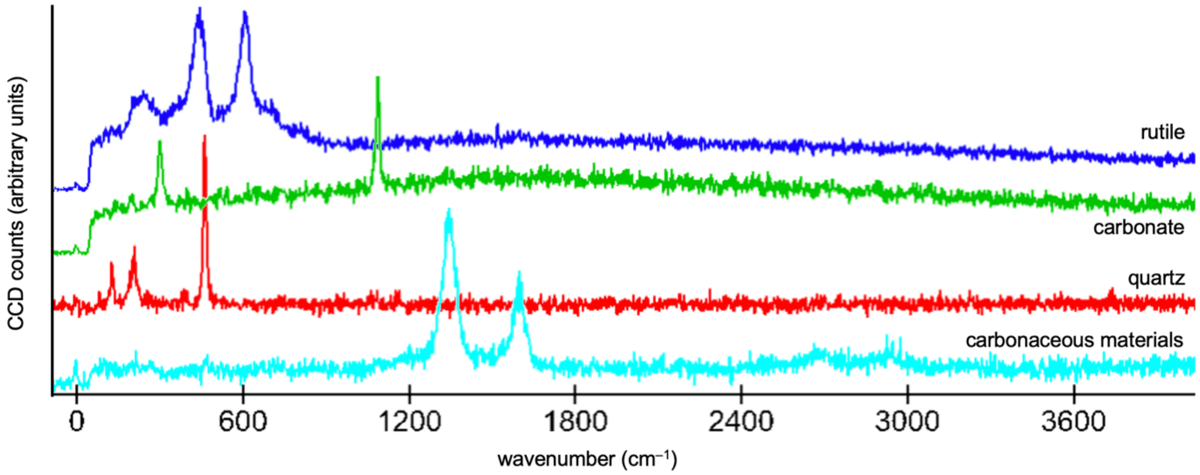

Figure S22. Representative Raman spectra maps for carbonaceous materials and major mineral phases.

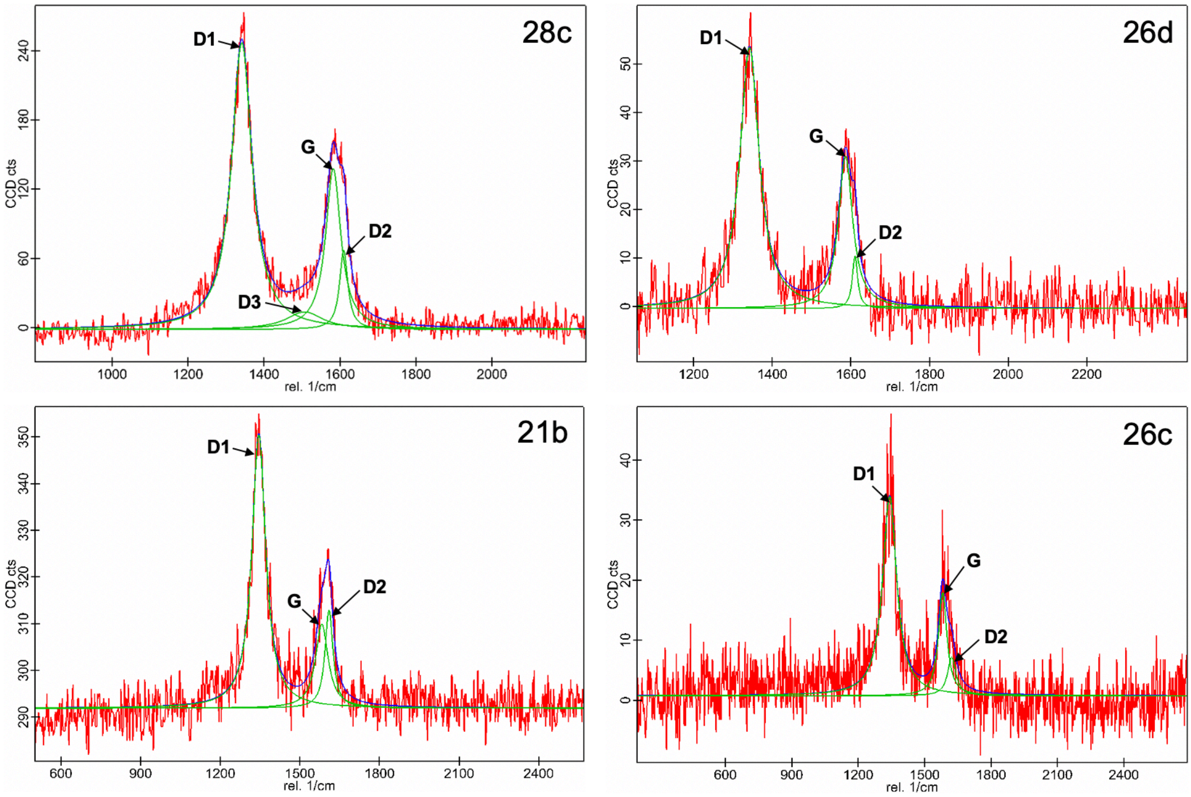

Figure S23. Spectral deconvolution of Raman D and G bands of carbonaceous materials, used to calculate peak thermal history of the samples.

Table S1. Raman geothermometry calculated peak temperatures.

**Sample**	**Peak temperature calculated within organic–phyllosilicate laminations**		**Peak temperature calculated within carbon-poor matrix**	
	Beyssac	*Kouketsu*	*Beyssac*	*Kouketsu*
28c	357 ± 50°C	321 ± 30°C	336 ± 50°C	363 ± 30°C
26d	330 ± 50°C	285(?) ± 30°C	356 ± 50°C	335 ± 30°C
21b	349 ± 50°C	343 ± 30°C	389 ± 50°C	354 ± 30°C
26c	330 ± 50°C	313 ± 30°C	332 ± 50°C	314 ± 30°C

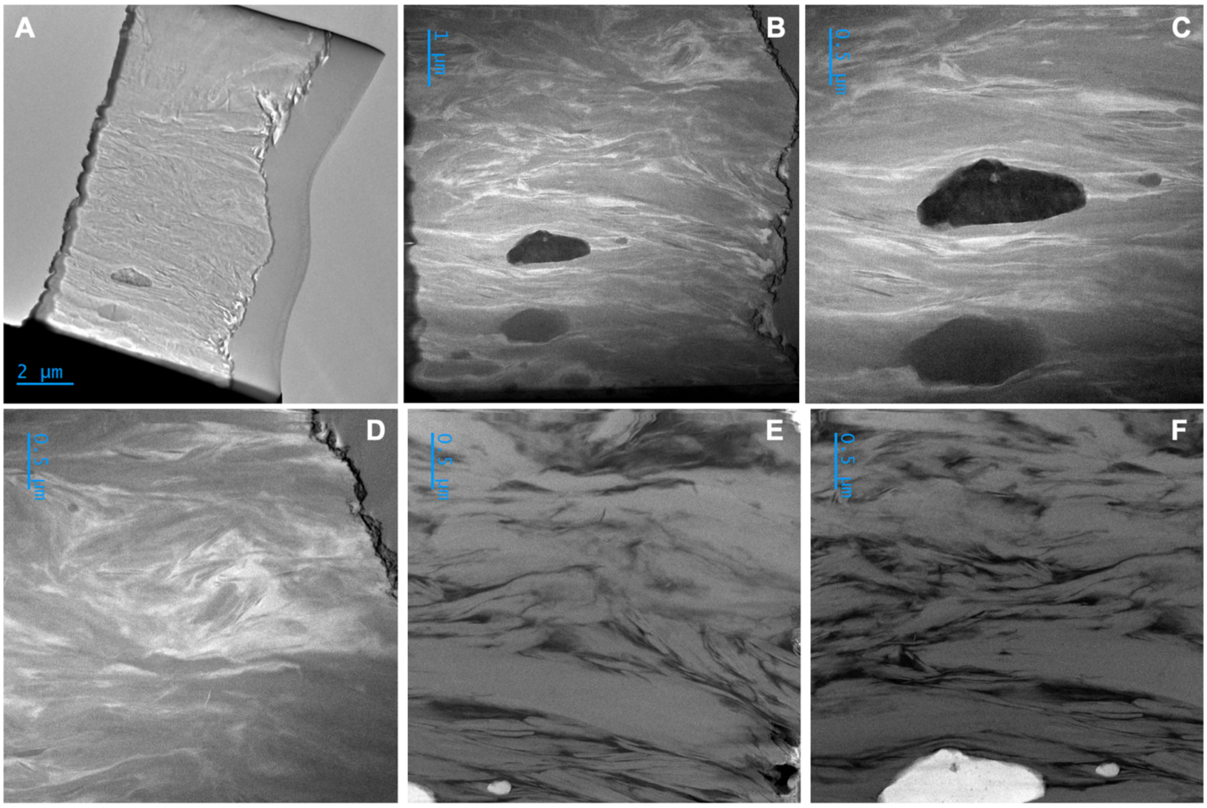

Figure S24. TEM images of ultrathin FIB-milled section from **s**ample 28c. A) TEM image of FIB section. B–D) High-angle annular darkfield (HAADF) images showing laminations of organic materials (white) intercalated with elongate phyllosilicate minerals (light grey) and heavy mineral phases (dark grey–black). See main text for details. E–F) Brightfield images showing laminations of organic materials (black) intercalated with elongate phyllosilicate minerals (dark grey) and heavy mineral phases (white).

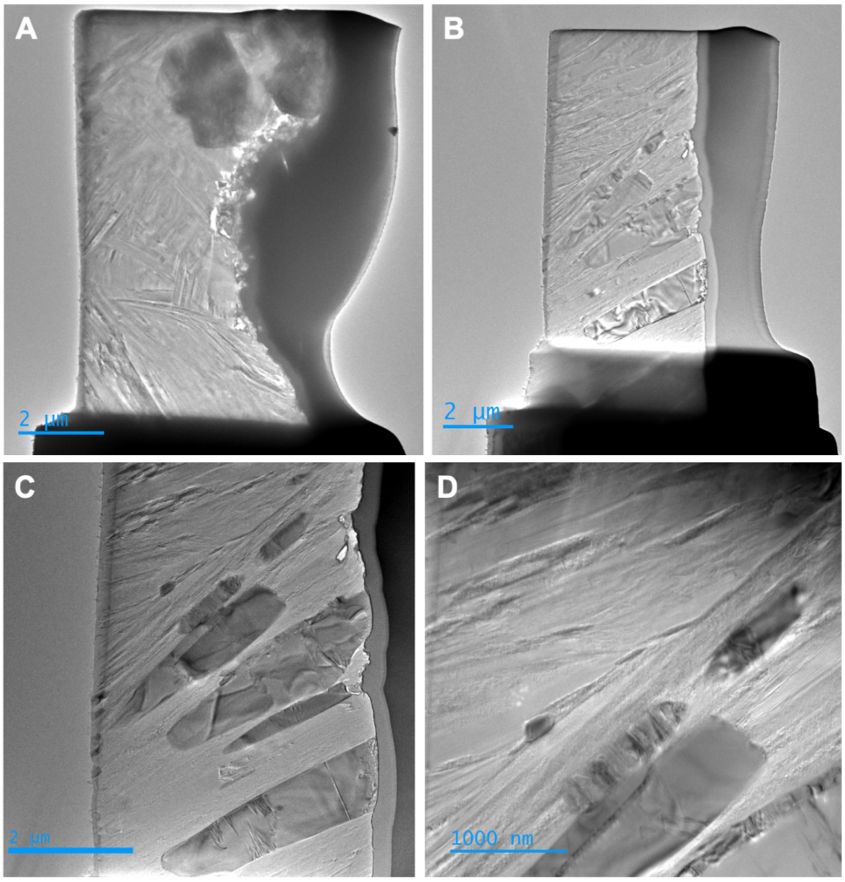

Figure. S25. A–D) TEM images showing FIB sections extracted from sample 26d.

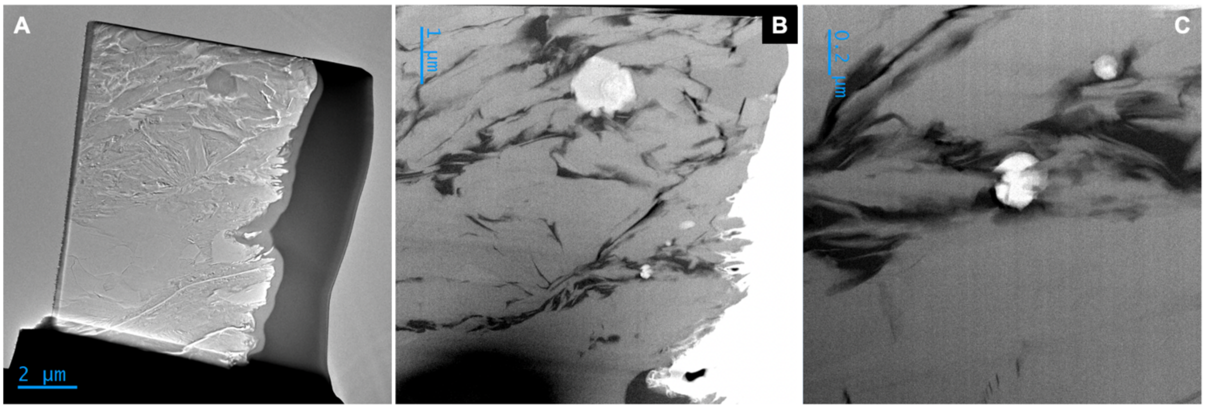

Figure S26. TEM images of FIB section extracted from sample 21b. A) TEM image showing entire FIB section. B–C) Brightfield images showing laminations of organic materials (black) intercalated with elongate phyllosilicate minerals (dark grey) and heavy mineral phases (REE phosphates, white).

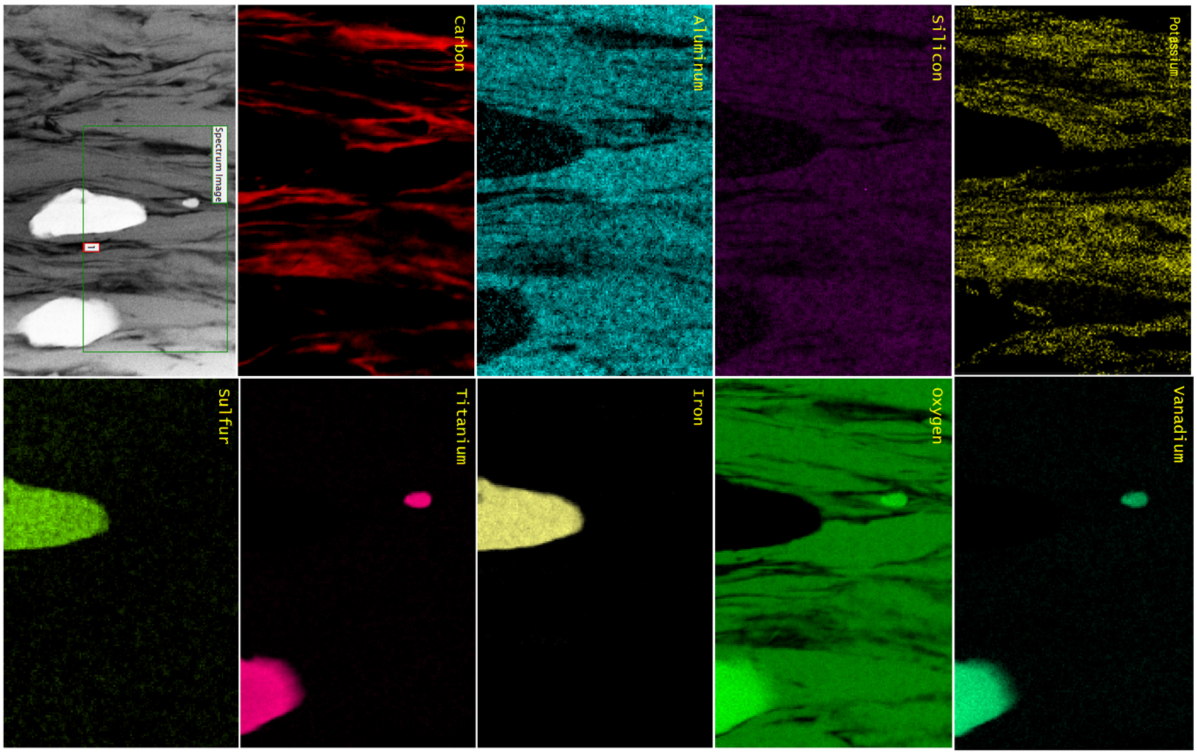

Figure S27. TEM imaging and EELS analyses of ultrathin FIB-milled section. EELS maps were obtained for C, Al, Si, K, S, Ti, Fe, O and V. Sample 28c.

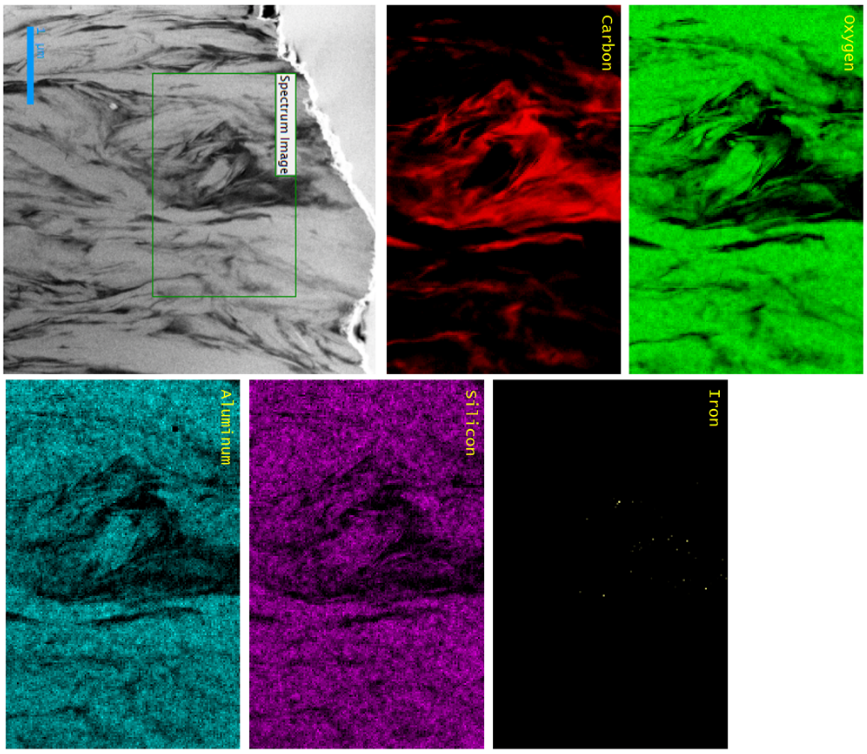

Figure S28. TEM imaging and EELS analyses of ultrathin FIB-milled section. EELS maps were obtained for C, O, Al, Si and Fe. Sample 28c.

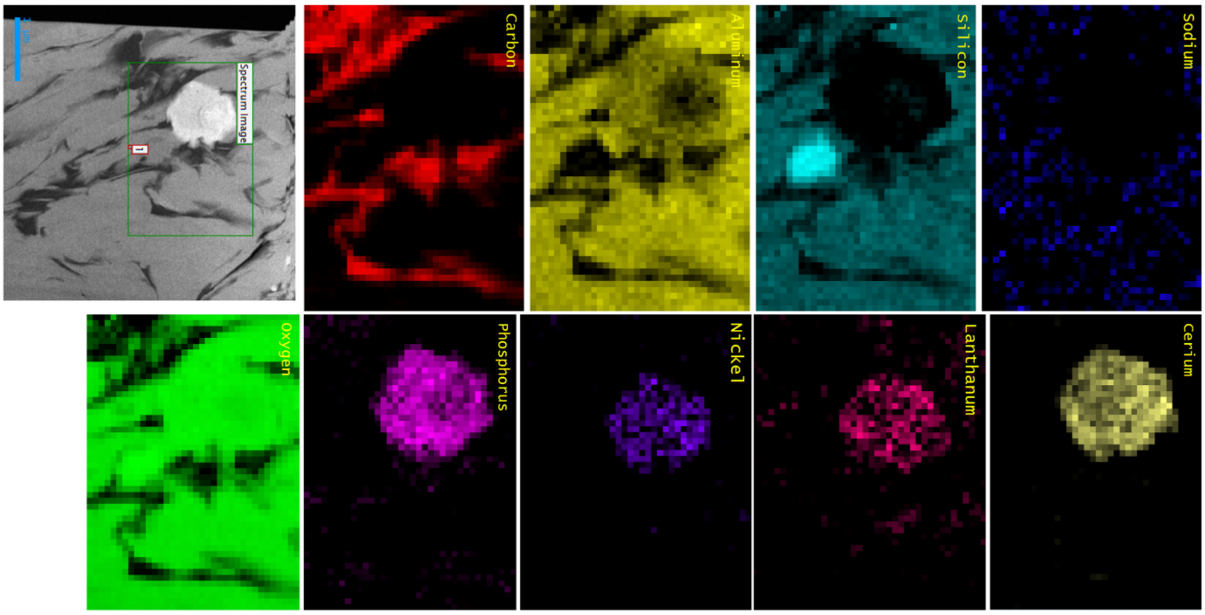

Figure S29. TEM imaging and EELS analyses of ultrathin FIB-milled section. EELS maps were obtained for C, Al, Si, Na, O, P, Ni, La and Ce. Sample 21b.

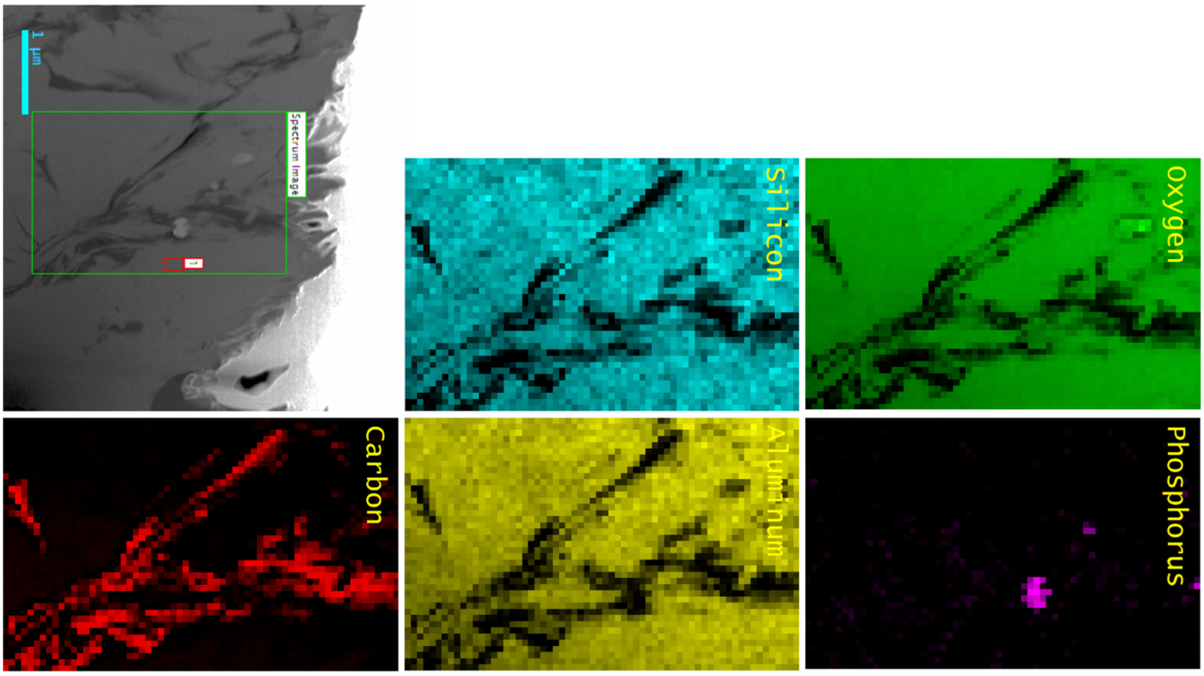

Figure S30. TEM image and EELS analyses of ultrathin FIB-milled section. EELS maps were obtained for C, Al, Si, O and P. Sample 21b.

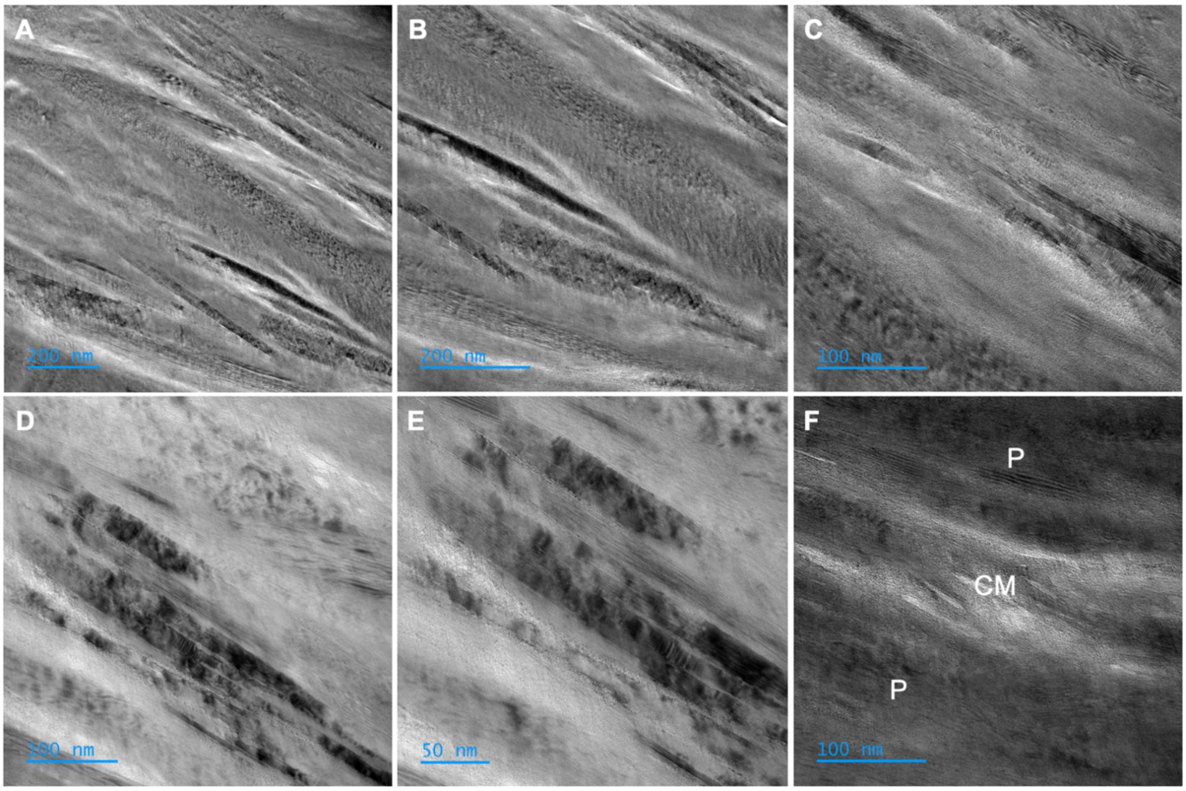

Figure S31. A–F) HRTEM images at increasing magnification showing the nanoscale intercalation of amorphous organic materials (white) and phyllosilicates (grey–black). Panel F shows clear interlayering of amorphous organics (CM) between well-oredered phyllosiciliate particles (P). Sample 28c.

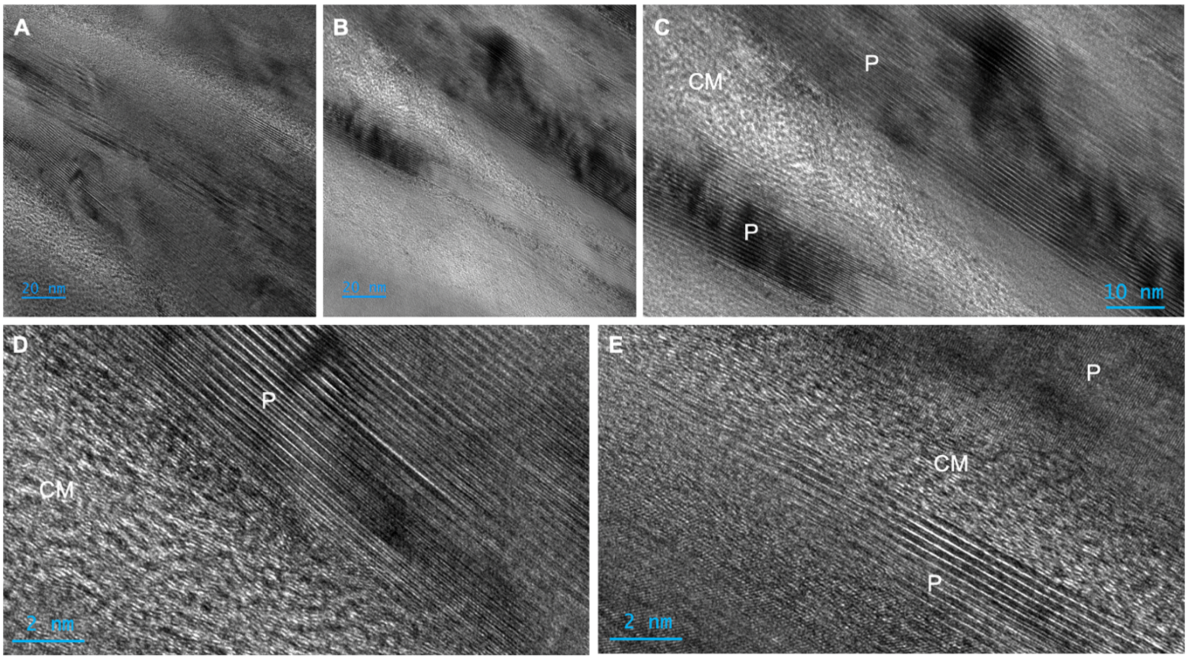

Figure S32. A–E) HRTEM images at increasing magnification showing the nanoscale intercalation of amorphous organic materials (white) and phyllosilicates (grey–black). B–E show clear interlayering of amorphous organics (CM) between well-ordered phyllosiciliate particles (P). Sample 28c.

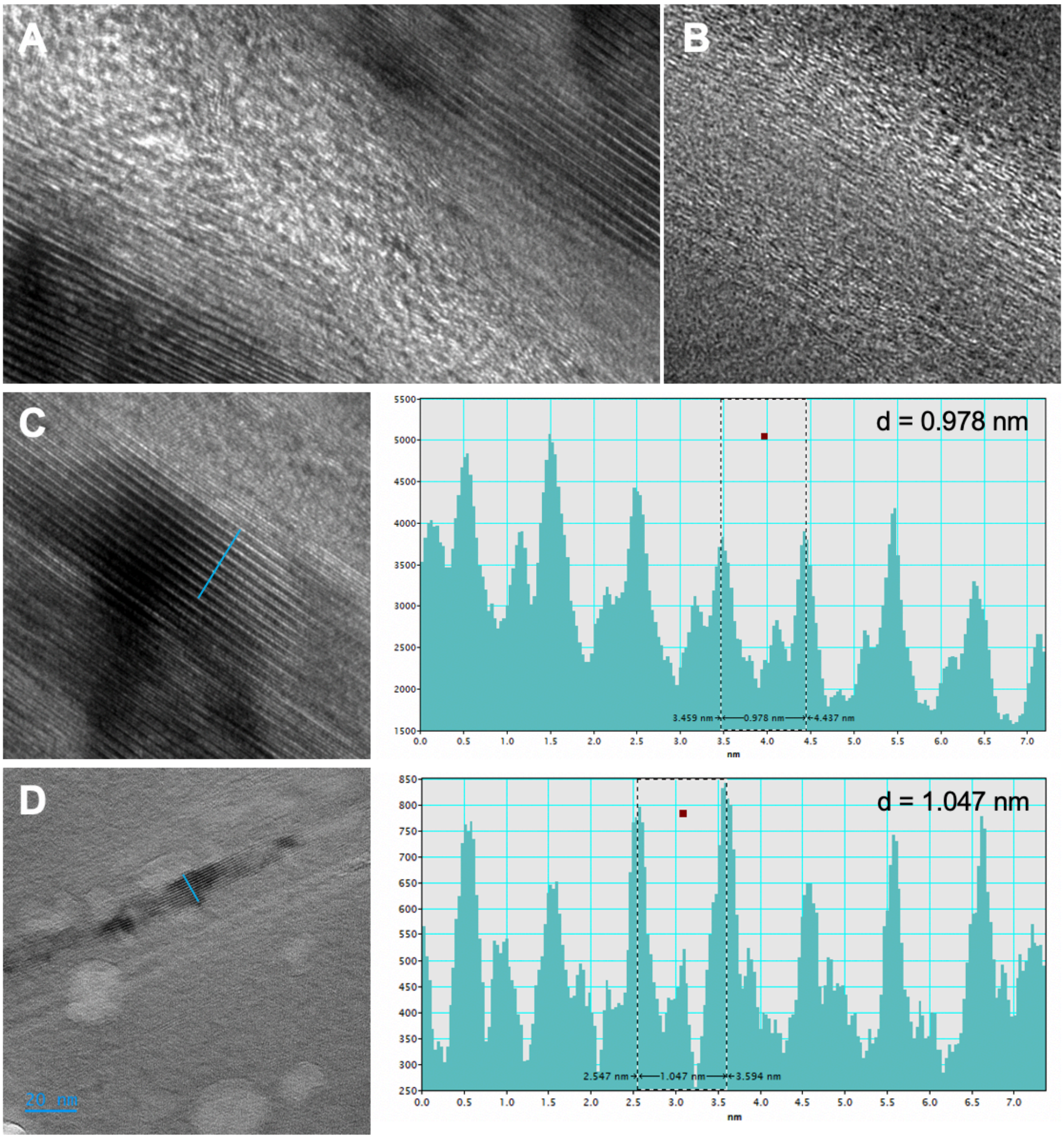

Figure S33. A–B) HRTEM images showing the nanoscale intercalation of amorphous organic materials (white) and phyllosilicates (grey–black). C–D) Calculation of interlayer spacing of phyllosilicates in sample 28c (C) and sample 26d (D).

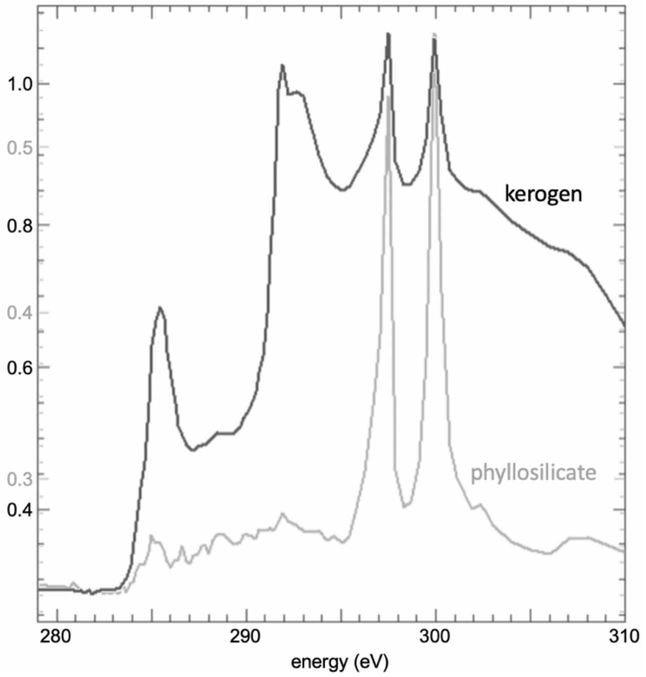

Figure S34. Representative STXM spectra from sample 28c. Selected STXM spectra are typical of organic materials (kerogen) with intense fine-edge structure between 283 and 292 eV (carboon *K*-edge), and phyllosilicates, with intense structure between 295 and 300 eV (potassium *L*-edge). Note the relative absence of carbon *K*-edge structure in the phyllosilicate spectrum. Potassium L-edge absorption in the kerogen spectrum occurs as a result of the extremely fine kerogen laminations, i.e. the analytical footpint includes a contribution from the surrounding phyllosilicates.

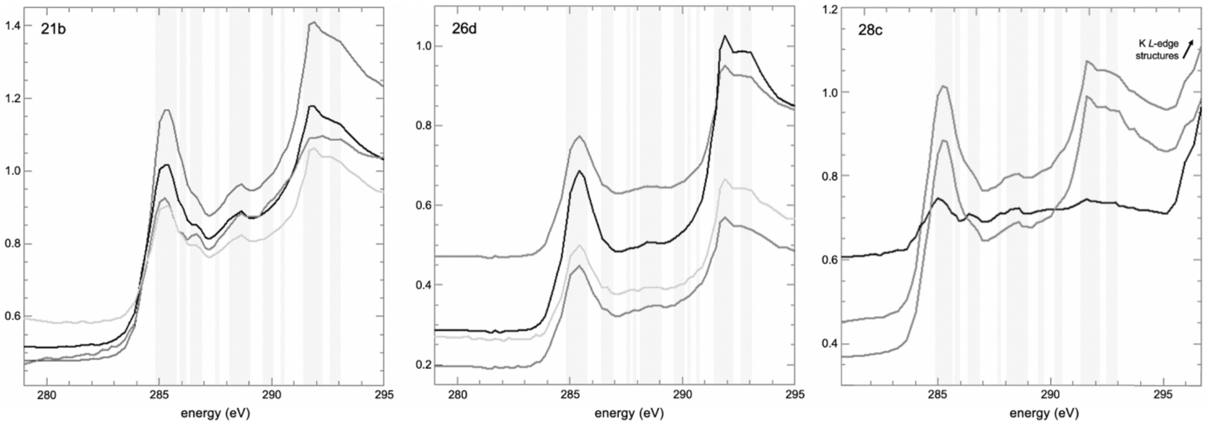

Figure S35. Representative STXM C *K*-edge (C-XANES) spectra of organic materials from samples 21b, 26d and 28c. See main text for the identification of absorption features.

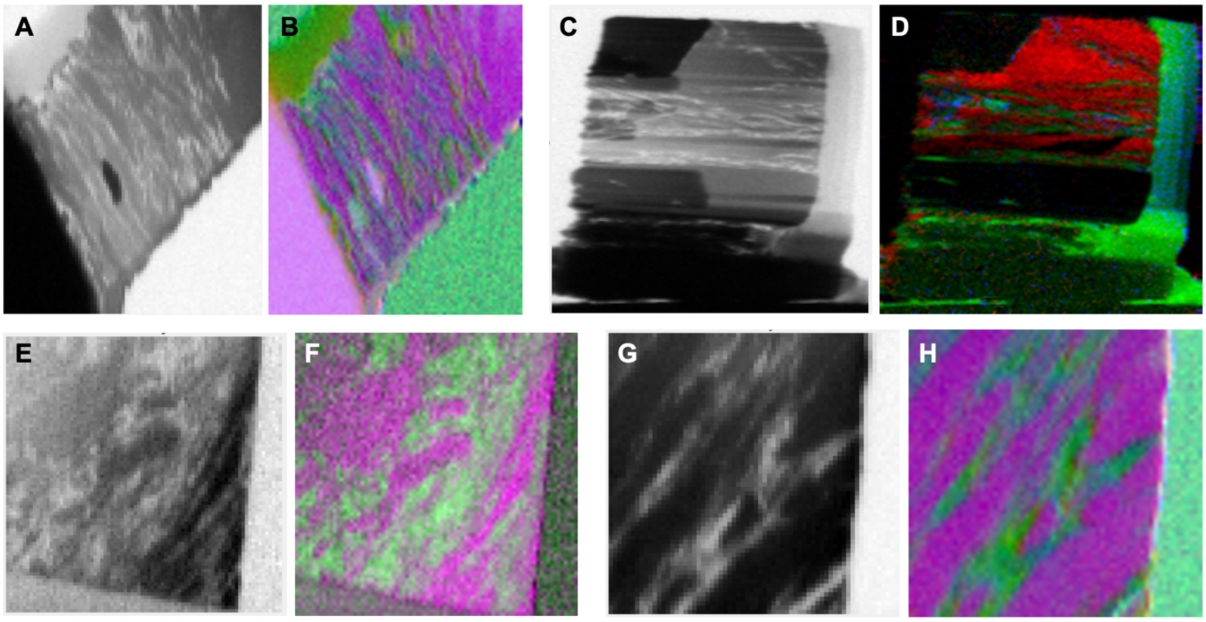

Figure S36. Representative STXM images and maps from A–B) sample 28c, C–D) sample 28c, E–F) 21b, and G–H) sample 26d. In A–B and E–H, green denotes carbon (peak at ~285 eV) and purple denotes phyllosilicates (peaks at ~298 and ~300 eV). In C–D, red denotes phyllosilicates (~298 eV) and blue and green denote the carbon peaks at ~285 and ~291 eV, respectively.

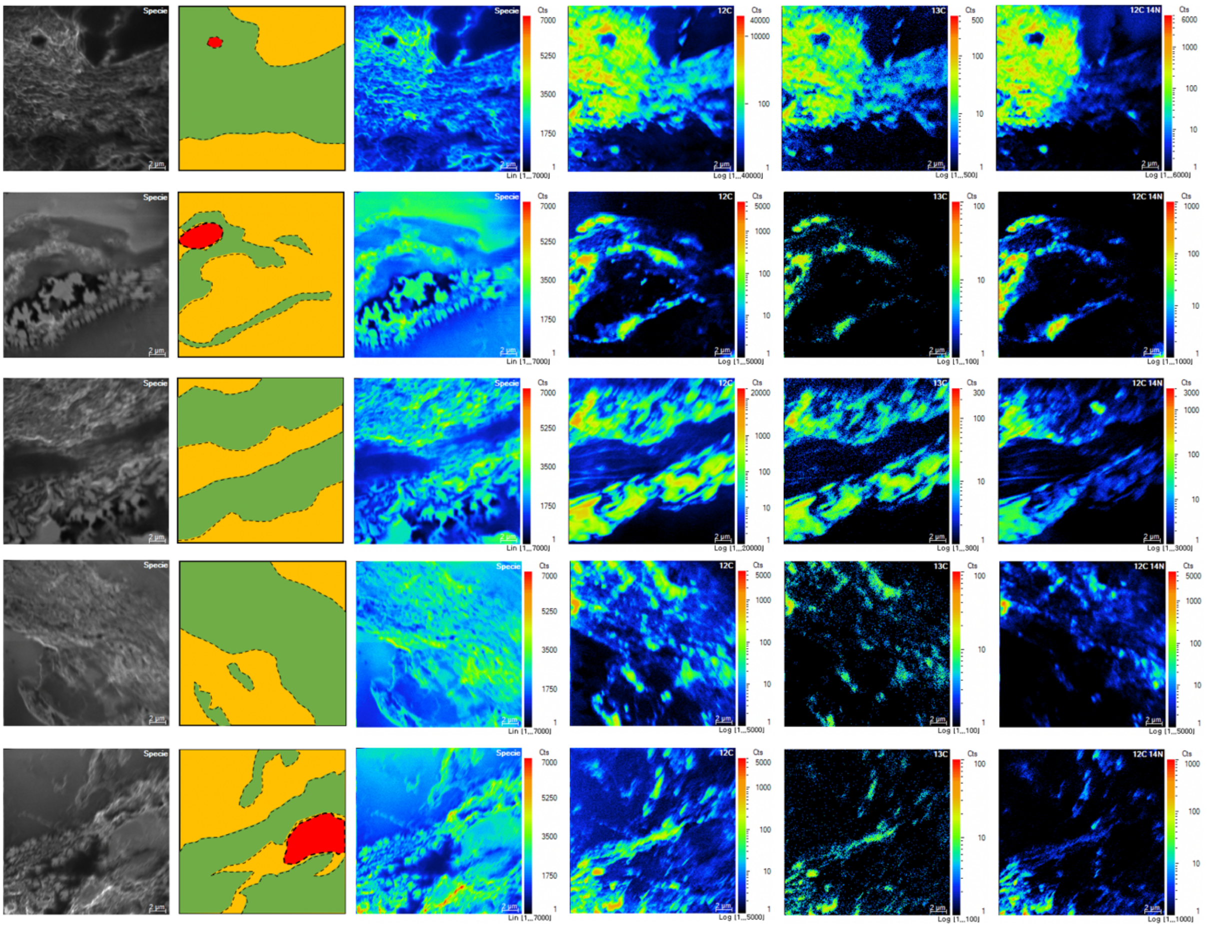

Figure S37. NanoSIMS ion mapping of microbial mat laminations. First column shows a schematic sketch of the mat lamination, where green denotes organic materials, amber denotes phyllosilicate and red denotes entrained heavy mineral particles. Second column shows cumulative ion species mapped, third column shows ^{12}C, fourth column shows ^{13}C and fifth column shows $^{12}C^{14}N$.
